# Vhavenda Herbal Remedies as Sources of Antihypertensive Drugs: Ethnobotanical and Ethnopharmacological Studies

**DOI:** 10.1155/2020/6636766

**Published:** 2020-12-11

**Authors:** Gundo Mudau, Samuel Odeyemi, John Dewar

**Affiliations:** Department of Life and Consumer Sciences, College of Agriculture and Environmental Sciences, University of South Africa, Johannesburg 1709, South Africa

## Abstract

Hypertension is a dominant risk factor for the development of cardiovascular, kidney, and eye diseases. In Africa, it increasingly leads to hospitalisation and a strain on the public health system. However, rather than modern medicine, African traditional healers are the first choice for most South Africans. Therefore, this study is aimed at gathering information on herbal remedies traditionally used for the treatment of high blood pressure in Vhavenda, South Africa, and comparing this information with reports in the literature regarding plants used to manage high blood pressure. An ethnobotanical survey was carried out in Vhembe district and its environs with 53 herbalists and indigenous people aged between 36 and 66 years from January to October 2019 using a semistructured questionnaire. The plants were collected with each respondent; they were authenticated and kept in herbarium. A total of 51 different plants were mentioned as being most commonly used for hypertension treatment. Of these, 44 plants were identified, with those from the Fabaceae family followed by plants from the Celastraceae family being commonly mentioned. Of these, the *Elaeodendron transvaalense*, *Tabernaemontana elegans*, *Elephantorrhiza elephantina*, and *Aloe vossii* were commonly cited species. According to the literature data, most of the identified plants are yet to be scientifically investigated for the treatment of hypertension, whereas only preliminary investigations have been carried out on other plants, suggesting that these preliminary investigations may have highlight promising antihypertensive activities *in vitro* that are indicative of their potential as antihypertensive drugs. Therefore, there is a need to scientifically investigate the antihypertensive potentials of these plants as a potential source of antihypertensive treatment and compounds.

## 1. Introduction

Hypertension is increasing at an alarming rate as a major public health concern after infection with the human immunodeficiency virus (HIV) and tuberculosis (TB). It is becoming one of the common cardiovascular diseases and a major health concern worldwide [1]. The two types of hypertension viz systolic and diastolic, graded as to patient blood pressure (BP): grade I or mild (BP 140–159/90–99 mmHg), grade II or moderate (BP 160–179/100–109 mmHg), and grades III and IV or severe (BP>180–210/110–120 mmHg) [2]. The exact causes of high blood pressure are unknown, but several risk factors such as family history, smoking, extensive use of alcohol, being overweight or obese, high sodium intake, high sugar intake, or lack of physical activity have been linked to the development of hypertension [3]. The incidence of hypertension has been reported mainly in individuals over 50 years in age, although there have been a few reports involving younger patients [4]. There are also reports that suggest that other conditions such as kidney failure, insulin resistance, atherosclerosis, cardiovascular diseases, and nervous system problems cause or exacerbate high blood pressure [5, 6]. There are different treatment regimens for hypertension, but these are associated with side effects, and hence, there is still a need for alternative treatment modalities. In this regard, different societies have their own systems in place to maintain and restore well-being, including traditional medicine that are of great importance as research has shown that their therapeutic properties are associated with secondary plant metabolites which treat the disease effectively with fewer or no side effects compared to the use of synthetic drugs [7].

A recent report suggests that more than three quarters of the world population depends on traditional medicine [8]. In Africa, the use of traditional medicine is popular due to trust and belief in its efficacy and dissatisfaction shown towards modern medicine [9]. Traditional medicine was in existence in Africa long before western medicine, and it is believed to link with “ubuntu,” connecting the patient to the land and to embrace nature [10]. Thus, traditional medicine is an integral and important part of the African heritage with this system developed by the society and passed on from one generation to the next in various forms [11, 12].

In Africa, traditional healers are believed to heal the physical and psychospiritual unwellness of an individual, reflecting a holistic approach to healing and treatments [9, 13]. This has led to a dependence on traditional healers such that they provide health services to over 80% of the population in rural communities due to their accessibility and affordability, and they have become the first choice for treatment for many people [14–16]. The relatively small number of health facilities and the associated delays in processing and treatment have influenced rural communities in their choice of traditional medicine rather than modern health care [10, 17]. In Thailand, the government has developed a healing system called “traditional Thai medicine” involving a health policy designed to reduce the use of expensive modern medicines that is linked to a scientific approach of 4-year curricula for training programs that culminate in a bachelor's degree [18]. Zimbabwe has also taken the initiative where they have their own Traditional Medical Practitioner Act which integrates traditional and modern medicines [19]. The South African government is attempting to close the gap between modern medicine and traditional medicine by providing complementary and alternative health services [20, 21]. In 2007, the South African government passed the Traditional Health Practitioner Act No. 22 so that in May 2014, sections of this Act provided more autonomy to the Traditional Health Practitioner Council of South Africa [22–24]. In light of these developments, this study is aimed at documenting indigenous knowledge on the herbal remedies used by the people of Vhembe District, Thulamela, South Africa, for the treatment of hypertension.

## 2. Methods

### 2.1. Ethnobotanical Survey

#### 2.1.1. The Study Area and Population

**Figure 1:**
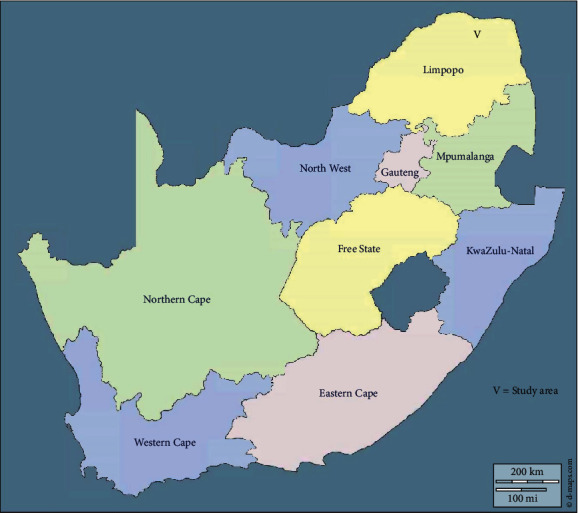
Study area: Vhembe district located in Limpopo province, South Africa.

Vhembe is one of the five districts in the Limpopo Province, South Africa (SA), located in the far northern part of South Africa sharing borders with Zimbabwe and Mozambique. It covers a surface area of 25,596 km^2^ with a population of 1,393,949 in 2016 (Figure 1), is a predominantly rural, cultural hub, and is a catalyst for agricultural and tourism development [25, 26]. The Vhembe district consists of three ethnics groups with Thohoyandou as the capital. It is the former Tsonga homeland of Gazankulu with Hlanganani and Malamulele. It has a population of 800,000 Venda-speaking, 400,000 Tsonga-speaking, and 27,000 Northern Sotho-speaking citizens [27]. Interviews with participants in this study were conducted at Thulamela municipality, Thohoyandou town which has a population of 618,462 and a growth rate of 0.62% (2001-2011) [27]. The study area falls into the category of villages with a high prevalence of hypertension.

#### 2.1.2. Ethics Approval

Consent to enter Tshififi village was obtained from the headman of the Tshikalange Tribal Authority. This allowed the proposed study to proceed within the jurisdiction of Tshififi and nearby areas. In practice, the respondents were each provided with a consent form that was approved by the University of South Africa's Ethics Committee (REC Reference No. 2018/CAES/146) before the study was explained to them. Each respondent who agreed to participate in the study then signed the consent form before the interviews were conducted in the knowledge that their anonymity was assured. Collected plant samples were authenticated at the Horticulture center, University of South Africa, Science Campus, and the voucher specimen was deposited.

#### 2.1.3. Data Collection

Since high blood pressure is a common health problem in the study area, the traditional healers were not asked for information on their diagnostic criteria. The survey was conducted during January and October 2019 and involved conducting face-to-face interviews with each respondent—in the local dialect where the respondent answered 12 open-end questions so that the researcher could collate demographic details of the traditional healers as well as the plants that they used to manage hypertension. The demographic information about each respondent included their age, gender, educational background, and locality. The information about the plants includes the vernacular names of the plants, parts used, methods of preparation of the recipes, route of administration, dosage, duration of treatment, and the management of other diseases for which the plants are used. The study relied on the recommendations of the headman, who identified the traditional healers and other qualified respondents.

#### 2.1.4. Plant Collection and Identification

A good rapport was established between the researcher and the respondents and with their assistance; plant species were collected during several visits over the course of the study. The selected respondents were those often-assisting traditional healers in plant collection from their natural habitat. A broad approach was used to correctly identify collected plant materials. This involved (i) comparison against samples in the Unisa Herbarium, (ii) against data from the literature, and (iii) consultation with botanists from within the Unisa College of Agriculture and Environmental Science (CAES) laboratories, the Unisa Department of Life and Consumer Sciences, and the Unisa Horticulture Centre. Consulted data bases included the PlantZAfrica database (http://pza.sanbi.org/), SANBI infobases (https://www.sanbi.org/resources/infobases/), and Vhenda inventory [28]. The voucher specimens of all the collected plant species were deposited in the Herbarium at the University of South Africa, Science Campus.

#### 2.1.5. Data Processing and Analysis

Data acquired from the questionnaire were uploaded onto a Microsoft Excel (365) spreadsheet and analyzed using both descriptive and inferential statistics. Percentages were used to analyse the respondents' sociodemographic data, and the relative frequency of citation (RFC) was used to determine the relative use of the plants.

*(1) Relative Frequency of Citation (RFC)*. This was calculated using the formula:

(1) $$\begin{array}{c} \text{RFC}=\frac{\text{Fc}}{N}, \end{array}$$

where Fc is the number of respondents who cited a species and *N* is the total number of the respondents. The RFC was used to determine the importance of a particular plant species (0 < RFC < 1).

### 2.2. Literature Review

ScienceDirect, PubMed, and Google Scholar databases were used to compare the literature reporting on medicinal plants showing antihypertensive activity against the data obtained in the survey. This was carried out using keywords (antihypertensive plants, ethnobotanical survey, medicinal plants, ethnomedicine, ethnobotany, herbal medicine, and treatment of hypertension). To obtain information on plants used in South Africa, the word “South Africa” was inserted and combined with the different keywords as indicated earlier.

## 3. Results

### 3.1. Ethnobotanical Survey

#### 3.1.1. Demographic Information of the Participants and Their Knowledge of Hypertension

A total of 60 respondents were approached to participate in this study, and of these, 53 agreed to participate including 23 traditional healers. As shown in the sociodemographic data of the participants (Table 1), the participants were based across 11 villages, 47.2% of the participants were males and 52.8% were females. All of the participants spoke Tshivenda, and most worship the ancestors (92.5%). The majority of the participants (43.4%) were within the age range of 56 to 66 years suggesting that the older age groups are the custodians of traditional knowledge. About forty percent (39.6%) of the participants had primary education, 47.2% secondary education, 1.9% tertiary education, and 11.3% had no formal education. None of the participants in this study was employed, and with the exception of one healer who used eight plant species and another who used 12 plant species, all of the participants used up to six plant species to treat hypertension. All the traditional healers that participated in this survey got their trainings through family knowledge particularly from ancestors.

#### 3.1.2. Diversity of Plants Used for the Treatment of Hypertension

**Figure 2:**
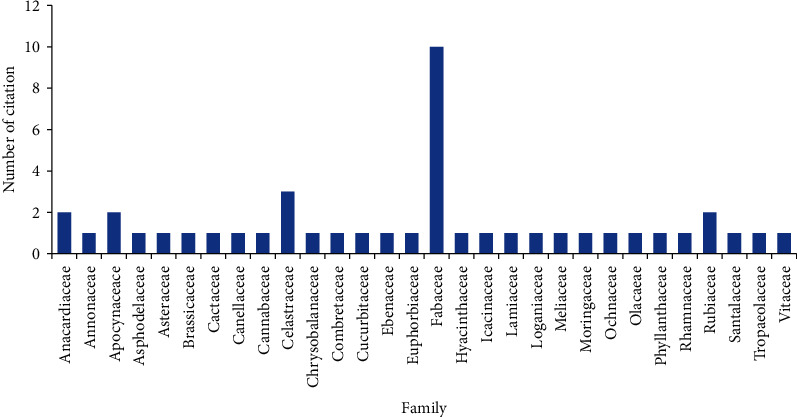
Distribution of use by traditional healers of plant families for the treatment of hypertension in the Vhembe district.

**Figure 3:**
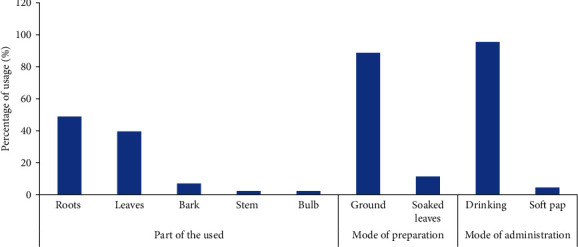
Plant parts as well as mode of preparation and administration of plants for use in treating hypertension.

The information on the medicinal plants used for traditional management of hypertension is presented in Table 2. A total of 51 plants species belonging to 30 families were reported as part of the hypertension treatment program in this study. The family distribution is shown in Figure 2. Members of the Fabaceae family were most commonly mentioned (10 times) followed by members of the Celastraceae family (3 times). Most of the plant parts used in the treatment decoction involved the roots, leaves, stems, and/or a combination of these parts (Figure 3). A decoction was prepared by drying, crushing, and soaked the plant part in water before a teacup of decoction was orally administered two or three times a day, while the majority (49%) of the respondents use the roots followed by the leaves with 40% usage (Figure 3).

#### 3.1.3. Frequently Collected Plant Species

The RFC value of each reported medicinal plant species was calculated and summarized (Table 2). The plants with 50% or more citations (RFC ≥ 0.5) were considered to be relatively important plants. In total, 3 plants were cited frequently by the respondents: Mukuvhazwivhi/Mulumanamana (*Elaeodendron transvaalense*), Muhatu (*Tabernaemontana elegans*), and Gumululo (*Elephantorrhiza elephantina*) with RFC values of 0.71, 0.52, and 0.52, respectively.

### 3.2. Analysis of Literature Review

From the review of literature, 62 families comprising to 139 plant species were reportedly used for the treatment of hypertension and related symptoms (Suppl. Table 1). The Asteraceae (*n* = 16) is the most commonly reported family, followed by the Fabaceae (*n* = 9), Rutaceae (*n* = 8), Anacardiaceae (*n* = 7), and Lamiaceae (*n* = 7) with the indicated number of plant species, respectively. The plants that were frequently cited in the literature are *Psidium guajava* L., *Catharanthus roseus* (L.), *Citrullus lanatus* (Thunb.), *Agave americana* (L.), *Hypoxis hemerocallidea* (Fisch.), *Musa acuminata*, *Clausena anisata* (Willd.), and *Ruta graveolens*.

#### 3.2.1. Comparative Analysis of the Ethnobotanical Survey with Literature Data

Comparing the antihypertensive plants in the ethnobotanical research with data from the literature revealed that 14% have been reported from the medicinal plants in the survey as antihypertensives. Furthermore, there are similarities between the ethnobotanical survey and data from the literature in terms of the most frequently cited families. The Fabaceae is the dominantly represented family whereas the most frequently reported plant is the *C. sativa* both in the survey and literature data. In contrast, 88% of the plant species identified in the present survey have not been reported previously in South Africa as antihypertensive plants. These newly reported plants include *Elaeodendron transvaalense*, *Tabernaemontana elegans*, and *Elephantorrhiza elephantina*.

#### 3.2.2. Plant-Derived Compounds Reported for Antihypertensive Activity

A quick summary obtained from the literatures clearly identified different classes of compounds. Some of these compounds have been evaluated for their antihypertensive activities using *in vitro* or *in vivo* assays belonging to different classes such as phenolics, flavonoids, glycosides, alkaloids, saponins, tannins, triterpenes, and peptides (Suppl. Table 1). Phenolics (*n* = 44) are the most commonly reported group of compounds identified with antihypertensive activities, followed by flavonoids (*n* = 31) and alkaloids (*n* = 27).

#### 3.2.3. Reported Mechanisms of the Herbal Remedies and Extracts towards the Alleviation of Hypertension

In the literature, most of the plants used for the management of hypertension carry out their antihypertensive activity through the inhibition of angiotensin-converting enzyme (ACE), reduction of oxidative stress, vasorelaxation via the nitric oxide-guanylyl cyclase pathway, and a prostaglandin-mediated mechanism as well as anti-inflammatory activities. Other reported mechanisms include the activation of the ATP-sensitive potassium channel, lowering of systolic blood pressure, EDRF-dependent or -independent pathways, endothelium-dependent vasorelaxation, a *β*1 agonist effect and direct vasoconstrictive effect, lowering left ventricular systolic pressure, reduction of systemic blood pressure and heart rate, inhibition of oxytocin-induced contraction, nitric oxide and angiotensin II-like activities, redox-sensitive phosphorylation of eNOS via the PI3-kinase pathway, and inhibition of Na+ and K+ reabsorption. Supplementary Table 1 shows that 24 plants inhibit ACE only, and 38 plants possess both antioxidant and anti-inflammatory activities, while 59 plant species have not been investigated for their mechanism of action.

## 4. Discussion

### 4.1. Demographic Information

As there were more females than males interviewed in the current survey, the predominance of women in relation to men can probably be ascribed to the involvement of men in other fields of work or the interview period such as when men were not at home. This is in agreement with the findings of a comparable study conducted in the Western Cape, South Africa [1], that reported a higher proportion of female over male respondents. The higher number of females to males in this study is similar to previous reports [1, 102, 103], but some authors also provided contrasting reports by suggesting that parents usually prefer to transfer indigenous knowledge to boys [104]. It will be fair to say that not all the respondents are traditional healers, so women being traditionally caretakers of the family's health may have impacted their knowledge on medicinal plants that exceed those of men. However, the predominance of women to men as the custodian of traditional knowledge varies according to the population under investigation in terms of their sociocultural characteristics. The higher percentage of ages between 55 and 66 years corroborates with previous reports suggesting that mainly adults and older people practice traditional medicine [103, 105, 106]. This study clearly shows the persistent gap in knowledge of herbal practice between the younger and older generations, suggesting the urgent need for documentation of this invaluable knowledge. Interestingly, the high education level of the informants and traditional healers in this study is enough to encourage the documentation of this knowledge or practice. This contrasts with reports that most traditional practitioners in Nigeria involved in maternal healthcare have no formal education [107].

### 4.2. Medicinal Plants Used in the Treatment of Hypertension

Most of the respondents in the present ethnobotanical survey mentioned medicinal plants belonging to the Fabaceae, Celastraceae, and Rubiaceae families. In other reports on the medicinal plants used for the treatment of hypertension, members of these families are often reported alongside with the Asteraceae and Rutaceae for use in the phytotherapy of various diseases including hypertension [50, 108–110]. In this study, the regular mention of the Fabaceae family is in agreement with previous reports on the plants used to manage hypertension [111–113]. This study also confirmed that the Fabaceae family is one of the plant families that is highly represented in the study area. The higher percentage usage of the roots and leaves is not uncommon and has been reported to be the preparation of herbal recipes in many other traditional medicines [114]. The reported findings in this study on the high frequency of leaves' use could be related to their visibility and ease of collection. The preparation of the leaves and roots in ground form for drinking is in agreement with surveys carried out in Ethiopia [115], Nigeria [116], Cameroon [117], and South Africa [108] suggesting that this is a common practice in traditional medicine. Most traditional healers may consider medicines that are milled in a mortar to a powder to be more efficient, as the powdered form of the plant material enhances the extraction of the active ingredients. Improved extraction efficiency from a powder may be due to a specific increase in the surface area of powdered particles that improves extraction of the medicinally important plant compounds. The predominance of trees to shrubs and herbs is similar to the survey in Botswana [118] and contrasts with the results of a survey in parts of the Eastern Cape province of South Africa where the commonly used plant is a herb [119]. To the best of our knowledge, *Elaeodendron transvaalense* and *Tabernaemontana elegans* frequently used in this study have never been reported in an ethnobotanical survey or investigated for the treatment of hypertension. However, *Elephantorrhiza elephantina* is part of the treatment regimen used by the Bapedi people for treating hypertension [120]. *Elaeodendron transvaalense* is also used in traditional medicine by the Vhavenda people of South Africa in the treatment of stomach ailments, cancer, diarrhoea, coughs, herpes, skin infections, inflammations, rashes, HIV/AIDS, and other sexually transmitted diseases. A report has indicated the extraction from *E. transvaalense* of three triterpenoids lup-20(30)-ene-3*α*,29-diol, lup-20(29)-ene-30-hydroxy-3-one, and *Ψ*–taraxastanonol together with some polyphenols [52]. Extracts of the plant *Tabernaemontana elegans* are used as to wash wounds, treat heart and pulmonary diseases, chest pains, and cancer, and have been reported to contain monoterpene bisindole alkaloids, such as tabernaemontanine, dregamine, 16-epidregamine, tabernaelegantine C, tabernaelegantinine B, voacangine, and vobasine. In addition, the alkaloids from *T. elegans* have been reported to induce apoptosis in colon carcinoma cells and show antimicrobial activity [38, 121, 122].

### 4.3. Antihypertensive Herbal Medicine Preparations and Route of Administration

This current study on plants used to treat hypertension reports mainly on single herbal preparations. Although there are reports on monocomponent recipes in traditional medicine, multiherbal preparations have also been reported [50, 108, 120]. Furthermore, the reported medicinal plants reported here are commonly used in the management of other disease conditions such as diabetes, cancer, sexually transmitted infections, tuberculosis, fever, skin infection, and sexual problems [2, 50, 114, 115, 117, 118, 121, 123]. In this study, the preparation of the medicinal recipes was by decoction and administered orally.

### 4.4. Analysis of the Literature Data Compared with the Survey

A review of the literature for plants used to treat hypertension in South Africa identified a large number of medicinal species (*n* = 139), and some of these plants have also been reported elsewhere for the treatment of hypertension. In South Africa, the most frequently cited plants are *Psidium guajava*, *Catharanthus roseus*, *Citrullus lanatus*, *Agave americana*, *Hypoxis hemerocallidea*, *Musa acuminata*, *Clausena anisata*, *Ruta graveolens*, *Lantana camara*, *Trichilia emetica*, *Leonotis leonurus*, *Ballota africana*, *Momordica charantia*, and *Cannabis sativa* [124–129]. For instance, *Psidium guajava* with the highest citation is also commonly used in other parts of the world such as India, Mexico, Nigeria, and Spain for the management of hypertension [124, 125, 129]. Comparison of the survey results with the literature data shows that the majority of the medicinal plants reported from the survey have not been previously reported for the treatment of hypertension. Although they appear in the inventory of plants used by the people of Vhenda, these plants have not been identified as antihypertensives [28]. This study indicates the multipurpose usage of medicinal plants and the dominance of the Fabaceae family of plants as an essential component of traditional medicine. With more than 490 species, the Fabaceae family is the second largest family of medicinal plants currently being used in traditional medicine. The family has been reported to show different medicinal potentials including antioxidant, antidiabetic, antibacterial, cytotoxic, and antihypertensive properties [130, 131]. In parallel to the data reported in the current study, the possible mechanisms for the antihypertensive properties of the Fabaceae family may involve the PI3-kinase/PKB/Akt pathway, inhibition of ACE and/or antioxidant properties [130].

#### 4.4.1. Plant Species in the Survey Previously Investigated for Their Antihypertensive Properties

The antihypertensive activity of *Opuntia ficus*–indica Mill. was reported among Zulu medicinal plants where the aqueous leaf extract inhibited the activity of ACE *in vitro* [132]. Other investigations on *O. ficus*–indica have indicated antihypercholesterolemic, antihyperlipidemic, anti-inflammatory, and antioxidant activities perhaps due to the presence of phenolics and flavonoid compounds [133]. *R. caffra*, also known as quinine tree, is a fast-growing tree predominantly found in Africa. It is traditionally used for the treatment of hypertension, cough, stomach ailments, wounds, and diarrhoea [36]. Antihypertensive activity associated with this plant was shown by a reduction in the systolic and diastolic blood pressure in spontaneously hypertensive rats [134]. The high blood pressure lowering effect has been linked to the presence of reserpine [135]. The cultivation for food of *Cannabis sativa* L. also known as hemp has been limited due to the presence of the psychoactive compound (tetrahydrocannabinol). However, a peptide isolated from the hemp seed has been reported to show antioxidant and antihypertensive activities through the inhibition of ACE [136].

#### 4.4.2. Toxicity Report

One of the traditional healers confirmed the warning that there is a need to carefully consider the use of Senna plant seeds in traditional remedies as the seeds of *Senna occidentalis* could be lethal [137]. The oral administration to rats of *Elephantorrhiza elephantina* extract was reported to lead to a decrease in their respiratory rate [123]. However, a report [52] confirmed that the use of *Elaeodendron transvaalense* showed few side effects. A report indicated that the extracts of *D. sanguinea* induced cardiac glycoside poisoning in sheep [138]. Foetidin, isolated from member of the Cucurbitaceae, has been reported to be toxic to certain cell lines [52]. Crude methanol and dichloromethane extracts of *S. didymobotrya* roots were reported to be toxic after a period of 14 days, killing 80% of mice at a dose of 5000 mg/kg body weight with an LD_50_ of 1927 mg/kg [103].

## 5. Conclusion

Traditional knowledge is sacred and is jealously guided. As a result, the Vhavenda people of South Africa have developed their own traditional way of treating hypertension. The present survey documented medicinal plants belonging to 30 families that are used for the treatment of hypertension and other diseases. Since the traditional healers in the study area use combinations of plants to treat hypertension, the efficacy of the treatment may be due to an ability to treat a broad spectrum of conditions such as bacterial infection, malaria, oxidative stress, and inflammation. This efficacy, together with the beneficial interaction between the healer and patient, may result in a psychosomatic improvement in the patient that combines to reduce blood pressure. However, according to the literature review, most of these plants have not been reported or investigated for their antihypertensive activity. This study will assist in the identification of useful plants. Also, these plants need to be investigated and their bioactive compounds isolated, to contribute to the discovery of new, effective, and affordable antihypertensive drugs.

## Figures and Tables

**Table 1:** The sociodemographic information of the respondents.

Biodata	Sex		Religion		Age group (years)				Level of education				Income level	
	Females	Males	Ancestor worshipers	Christianity	36-45	46-55	56-66	>66	No formal education	Primary education	Secondary education	Tertiary education	Pensioners	Client based
Traditional healers	12	11	20	1	3	9	10	1	4	11	13	1	6	17
Other respondents	16	14	29	3	4	12	13	1	2	10	12	-	-	-
Percentage	52.8	47.2	92.5	7.5	13.2	39.6	43.4	3.8	11.3	39.6	47.2	1.9	26.1	73.9

**Table 2:** Ethnobotanical information of plants used by traditional healers to treat hypertension.

Family	Local name	Botanical name	Part(s) used/collected	Voucher number	Mode of preparation	Route of administration	RFC	Plant growth form	Relevant reported disease treated or use
Anacardiaceae	Muadaba/Mulivhadza	*Lannea schweinfurthii* Engl.	Roots	GVM-017	Ground	Drinking	0.33	Tree	High blood pressure, snake bite, diarrhoea, fever, and malaria [29]
Anacardiaceae	Munungumaswi	*Ozoroa reticulata* (baker f.) R. & A. Fern. subsp.	Roots	GVM-024	Ground	Drinking	0.09	Shrub	Diarrhoea, stomach pain, vaginal and oral candidiasis, malaria, aphrodisiac, and cholera [30]
Annonaceae	Muvhulavhusiku	*Xylopia odoratissima* Welw. Ex Oliv.	Roots	GVM-039	Ground	Drinking	0.09	Tree	Infertility, stomach ache, diabetes, abdominal ulcers, fever, epilepsy, and angina [31, 32]
Apocynaceace	Munadzi	*Rauvolfia caffra* Sond	Leaves	GVM-030	Ground	Drinking	0.24	Tree	Tumour, sexually transmitted infections, anxiety, psychosis, schizophrenia, insanity, insomnia, and epilepsy [33–36]
Apocynaceae	Muhatu	*Tabernaemontana elegans* Stapf	Roots	GVM-034	Ground & fresh leaves soaked	Drinking & added to soft porridge	0.52	Tree	Cancer, chest pain, tuberculosis, venereal diseases, wounds, and menorrhagia [37, 38]
Asphodelaceae	Tshikhopha/Tshikopa	*Aloe vossii* Reynolds	Leaves	GVM-002	Ground	Drinking	0.47	Shrub	No record
Asteraceae	Mushidzhi	*Bidens Pilosa* L.	Leaves	GVM-004	Ground	Drinking	0.09	Shrub	Fever, malaria, inflammation, hyperemesis gravidarum (morning sickness), wounds, intestinal worms, otitis, dysentery/bacillary dysentery, constipation, Colics, and cancer [39–41]
Brassicaceae	Muobadali	*Capparis tomentosa* Lam.	Roots	GVM-009	Ground	Drinking	0.28	Shrub	Headache, mental disorder, snake bites, chest pains, impotency, and barrenness [42, 43]
Cactaceae	Mudoro	*Opuntia ficus-indica* (L.) Mill.	Bark	GVM-022	Ground	Drinking	0.09	Shrub	Weight control, diabetes, hypertension, asthma, ulcers, rheumatic pain, wounds, and fatigue [44, 45]
Canellaceae	Mulanga	*Warburgia salutaris* (G.Bertol.) Chiov.	Leaves	GVM-037	Ground	Drinking	0.09	Tree	Bronchial infections, oral thrush, cystitis, coughs, colds, tuberculosis, influenza, sinus, and other respiratory complaints [46–48]
Cannabaceae	Mbanzhe	*Cannabis sativa* L.	Leaves and stem	GVM-008	Ground	Drinking	0.18	Tree	Sprue syndrome, sterility, impotency, diarrhoea, indigestion, epilepsy, insanity, colic pain, and diabetes [49, 50]
Celastraceae	Malambamapikwa	*Elachyptera parvifolia* (Oliv.) N. Hallé	Roots	GVM-013	Ground	Drinking	0.09	Shrub	Limited data
Celastraceae	Mukuvhazwivhi/Mulumanamana	*Elaeodendron transvaalense* R.H.Archer	Bark	GVM-014	Ground& fresh leaves soaked	Drinking	0.71	Shrub	Diabetes, coughs, diarrhoea, stomach ailments, herpes, and sexually associated diseases [51–53].
Celastraceae	Mukwatikwati	*Mystroxylon aethiopicum* (Thunb.) Loes.	Bark and root	GVM-040	Ground	Drinking	0.09	Tree	Haemorrhagic diarrhoea, infectious diseases, and magic [28, 54]
Chrysobalanaceae	Muvhula	*Parinari curatellifolia* planch*. ex Benth*	Roots	GVM-026	Ground	Drinking	0.24	Tree	Hypertension, diabetes and liver-related illnesses [55, 56]
Combretaceae	Mufhatelathundu	*Combretum zeyheri* Sond.	Roots	GVM-010	Ground	Drinking	0.09	Tree	Tumours or diarrhoea, hypertension, and even cancer [57]
Cucurbitaceae	Nyapiringuhule	*Momordica boivinii* Baill.	Roots	GVM-020	Ground	Drinking	0.09	Shrub	Spiritual ailments, stomach problem [58, 59]
Ebenaceae	Mukwatikwati	*Euclea linearis* Zeyh. Ex Hiern	Roots	GVM-016	Ground	Drinking	0.09	Shrub	Malaria [60]
Euphorbiaceae	Masunungule	*Croton gratissimus* Burch.	Leaves	GVM-011	Ground	Drinking	0.09	Tree	Coughs, chest complaints, coughs, fever, sexually transmitted diseases, skin care, and perfumery [61, 62]
Fabaceae	Muangaila	*Millettia stuhlmannii* Taub.	Roots	GVM-019	Ground	Drinking	0.09	Tree	Stomachache and protection of homesteads and properties [63]
Fabaceae	Muvhambangoma	*Albizia versicolor* Oliv	Leaves and roots	GVM-001	Ground	Drinking	0.09	Tree	Venereal diseases, coughs, joint pains, tapeworms, fever, diarrhoea, and sores [64–66]
Fabaceae	Mufhulu	*Burkea africana* Hook.	Leaves	GVM-007	Ground	Drinking	0.09	Tree	Headache, migraine, dizziness, pain, inflammation, thrush, tooth ache, heavy menstruation, abdominal pain, inflammation, and pneumonia [67, 68]
Fabaceae	Gumululo	*Elephantorrhiza elephantina* (Burch.) Skeels	Leaves and roots	GVM-015	Ground & fresh leaves soaked	Drinking	0.52	Shrub	Dysentery, diarrhoea, coughing, pneumonia, chest complaints, heart conditions, hypertension, stomach ailments, syphilis, infertility in women, waist pain in infants, fever, haemorrhoids, aphrodisiac, and emetic to mitigate the anger of the ancestors [69, 70]
Fabaceae	Muhataha	*Pterocarpus rotundifolius* (Sond.) Druce	Roots	GVM-028	Ground	Drinking	0.09	Tree	Anemia, venereal, kidney diseases, and fertility in cows [14, 71]
Fabaceae	Mutsheketsheke	*Senna didymobotrya* (Fresen). H.S Irwin & Barneby	Aerial parts	GVM-041	Ground	Drinking	0.19	Shrub	Fever, enteric problems, anthelmintic, and antifungal [72, 73]
Fabaceae	muḓuwaḓuwane	*Senna italica* Mill.	Leaf and root	GVM-042	Ground	Drinking	0.19	Shrub	Laxative, purgative, constipation, rheumatic, and intestinal disorders [74]
Fabaceae	Muyekeyeke	*Senna obtusifolia* (L.) H.S.Irwin & Barneby	Root	GVM-043	Ground	Drinking	0.19	Herb	Laxatives, treatment of scorpion stings, gingivitis, dysentery, diarrhoea, tremors, and for dark brown urine [75, 76]
Fabaceae	Mutsheketsheke	*Senna occidentalis* (L.) Link	Roots	GVM-032	Ground	Drinking	0.19	Shrub	Laxative, analgesic, expectorant, diuretic, anthelmintic, tuberculosis, gonorrhoea, urinary tract diseases, and liver diseases [77]
Fabaceae	Mukundulela	*Vigna vexillata* (L.) A.Rich.	Leaves	GVM-036	Ground	Drinking	0.09	Shrub	Food, cancer, cardiovascular diseases, and diabetes [78]
Hyacinthaceae	Tshiganama	*Drimia sanguinea* (Schinz) Jessop	Bulb	GVM-012	Ground	Drinking	0.09	Shrub	Candidiasis, common warts, condylomata acuminate, genital warts, syphilis, and yaws [79]
Icacinaceae	Galange	*Pyrenacantha kaurabassana* Baill.	Roots	GVM-029	Ground	Drinking	0.09	Shrub	Ulcers, diarrhoea, herpes, and HIV [80, 81]
Lamiaceae	Mukwatikwati	*Volkameria glabra* (E. Mey.) Mabb. & Y.W. Yuan	Leaf and root	GVM-044	Ground	Drinking	0.09	Tree	Limited information
Loganiaceae	Mukongovhoti	*Strychnos potatorum* L.f.	Leaves and roots	GVM-033	Ground	Drinking	0.24	Shrub	Inflammation, anemia, jaundice, gonorrhea, leucorrhoea, gastropathy, bronchitis, chronic diarrhoea, dysentery, strangury, renal and vesicle calculi, diabetes, burning sensation, dipsia, conjunctivitis, scleritis, ulcers, and some eye diseases [82, 83]
Meliaceae	Museranga	*Melia azedarach* L.	Leaves	GVM-018	Ground	Drinking	0.09	Tree	Antidiarrhoeal, deobstruent, diuretic, anthelmintic, and constipating [84]
Moringaceae	Muringa	*Moringa oleifera* Lam.	Leaves	GVM-021	Ground	Drinking & added to soft porridge	0.33	Tree	Diabetes, tuberculosis, fever, stomach aches, ear infections, skin infections, lithiasis, hypertension, microbial, fungal viral infections, hepatotoxicity, inflammation, and fever. [85, 86]
Ochnaceae	Mutavhatsindi	*Brackenridgea zanguebarica* Oliv.	Roots	GVM-005	Ground	Drinking	0.09	Tree	Cause sterility in adults, warding off evil spirits, and protection from lightning strikes [87, 88]
Olacaeae	Tshitanzwatanzwa	*Ximenia americana* L.	Roots	GVM-038	Ground	Drinking	0.09	Shrub	Antiseptic, measles, jaundice, and headaches [89]
Phyllanthaceae	Munzere	*Bridelia micrantha* (Hochst.) Baill.	Bark	GVM-006	Ground	Drinking	0.24	Tree	Gastrointestinal ailments, painful joints, retained placenta, diabetes mellitus, syphilis, prehepatic jaundice, tape worm abdominal pain, conjunctivitis, headache, scabies, bloody diarrhoea, dysentery, emetic, wound infection, coughs, threadworms, tonic for children, sore eyes, epigastric pain, relief of headache, purgative diarrhoea, and worms [90, 91]
Rhamnaceae	Munie	*Berchemia discolor* (Klotzsch) Hemsl.	Leaves	GVM-003	Ground	Drinking	0.24	Tree	Malaria [92]
Rubiaceae	Sulesule	*Paederia bojeriana* (A.Rich. Ex DC.) Drake	Leaves	GVM-025	Ground & fresh leaves soaked	Drinking	0.09	Tree	No record
Rubiaceae	Mutondo	*Pterocarpus angolensis* DC	Leaves	GVM-027	Ground	Drinking	0.24	Tree	Diarrhoea, heavy menstruation, nose bleeding, headache, stomachache, schistosomiasis, sores, and skin problems [93, 94]
Santalaceae	Mupeta	*Osyris lanceolata* Hochst. & Steud.	Roots	GVM-023	Ground & fresh leaves soaked	Drinking	0.28	Shrub	Candidiasis, venereal diseases, styptic effects on wounds, menorrhagia, and infertility [95, 96]
Tropaeolaceae	Bopa	*Tropaeolum majus* L.	Leaves	GVM-035	Ground	Drinking	0.09	Tree	Antidepressant, hypertension, constipation, asthma inflammation, and urinary tract infection [97–99]
Vitaceae	Mutumbulambudzana	*Rhoicissus tridentata* (L.f.) Wild & R.B. Drumm.	Roots	GVM-031	Ground	Drinking	0.09	Shrub	Helminths (worms) & inflammation, miscarriages, and diarrhoea [100, 101]

## Data Availability

All the data used to support the findings of this study are included within the articles, and the plant samples are available at the University of South Africa, Florida Campus.

## Supplementary Materials

Supplementary Materials Supplementary Table 1: ethnopharmacological details of reported medicinal plants used by traditional healers in South Africa.
