# Supplementary material for: Vhavenda Herbal Remedies as Sources of Antihypertensive Drugs: Ethnobotanical and Ethnopharmacological Studies
Source: Oxid Med Cell Longev. 2020 Dec 11;2020:6636766. doi: 10.1155/2020/6636766 (PMC7748911; doi:10.1155/2020/6636766)
Supplement: Supplementary Materials — Supplementary Table 1: ethnopharmacological details of reported medicinal plants used by traditional healers in South Africa. [file 6636766.f1.docx]

Supplementary Table 1: Ethno-pharmacological details of reported medicinal plants used by traditional healers in South Africa.

| Family | Plant species | Local name/common name | Bioactive Molecules | Toxicity | Mechanism of Action | Life form | Parts  used | Citations | References |
| --- | --- | --- | --- | --- | --- | --- | --- | --- | --- |
| Acanthaceae | *Asystasia gangetica* L | Isihobo (Z) | flavonoids, phenols, alkaloids, cardiac glycosides, tannins, and terpenoids | Cytotoxicity reported [1, 2] | inhibition of ACE and the ANG II receptors | Herb | L | 3 | [3–5] |
|  | *Justicia flava* (Vahl) Vahl | Impela (Z); geelgarnaalbos (A) | β-sitosterol, stigmasterol, campesterol, β-sitosterol-β-D-glucoside, and salicyclic acid. | NR | inhibition of ACE | Herb | L | 2 | [5, 6] |
| Agapanthaceae | *Agapanthus africanus* (L.) Hoffmg. | kleinbloulelei (A) | Spirostane, furostane, saponins and sapogenins | NR | inhibition of ACE | Herb | L,Rt | 2 | [7, 8] |
| Agaricaceae | *Agaricus bisporus* (J.E. Lange) Imbach | Ikhowe (Z) | peptides | NR | inhibition of ACE; reduction of serum total cholesterol level | mushroom | Fr | 2 | [9, 10] |
| Agavaceae | *Agave americana* L. | Mobepi/kgopha-ya-pala (P) | Saponins and sterols | Cytotoxicity reported | inhibition of ACE | Shrub | L | 6 | [7, 9–13] |
| Aizoaceae | *Mesembryanthemum crystallinum* L. | rosa de jericho (Eng.); brakslaai, brakvy (A) | resveratrol and catechin | NR | inhibition of ACE | Herb | L, S | 3 | [7, 14–16] |
|  | *Mesembryanthemum varians* Haw. | soutvygie (A) | NR | NR | NR | Herb | L, S | 1 | [7] |
|  | *Sceletium tortuosum* (L.) N.E.Br. | Tandtrekbos (A) | NR | Safe at 8 -25 mg once daily in healthy adults | NR | Herb | L, Rt | 2 | [7, 17, 18] |
| Alliaceae | *Tulbaghia acutiloba* Harv. | Ishaladi Lezinyoka (Z); wildeknoffel (A) | NR | NR | inhibition of ACE | Herb | B, Fl, WP | 1 | [19] |
|  | *Tulbaghia violacea* Harv. | wilde knoffel (A); utswelane (X); incinsini, (Z) | Saponins and sulphur compounds | NR | direct negative chronotropic effect; inhibition of ACE | Herb | Rh, B, L, Rt | 4 | [7, 20–22] |
| Amaranthaceae | *Achyranthes aspera* | Isinama (Z) | phenolics | NR | Antioxidant and anti-inflammatory activity | Herb | Rt, L | 2 | [23–25] |
|  | *Amaranthus dubius* Mart. ex Thell. | vowa (V); pigweed (E); misbredie (A) | ascorbic acid, β-carotene, alkaloids, tannins, saponins, and glycosides | NR | inhibition of ACE | Herb | L | 1 | [5, 25] |
|  | *Amaranthus hybridus* L. subsp. cruentus (L.) Thell. | Vowa, gango (V); red amaranth, common pigweed (E); gewone misbredie (A) | cardiac glycosides, steroids, flavonoids, and terpenoids | NR | inhibition of ACE | Herb | L | 1 | [5] |
| Amaryllidaceae | *Agapanthus africanus* | Ubani (Z) | spirostane | NR | inhibition of ACE | Herb | L, Rt | 2 | [7, 23] |
|  | *Allium sativum* L. | khanakhana (V); garlic (E); knoffel (A) | peptides, terpenoids, phenols, saponins and sulphur compounds | Safety profile, antifungal reported | antioxidant | Herb | Fl bud | 1 | [26, 27] |
|  | *Crinum macowanii* Baker | Umdube, Umnduze (Z); Boslelie (A) | alkaloids | genotoxic, mutagenic, and nematicidal | Lowering of systolic and diastolic BPs, as well as mean arterial blood pressure | Herb | B, L, WP | 2 | [28, 29] |
|  | *Gethyllis* L. | Koekoemakranka (A) | NR | NR | NR | Herb | Sd, B, pod | 2 | [26, 30] |
| Anacardiaceae | *Ozoroa engleri* R. Ferm & A. Ferm | Isfico (Z) | NR | Anti-pathogenic activity reported | NR | Tree | Rt | 1 | [31, 32] |
|  | *Protorhus longifolia* (Bernh.) Engl. | red Cape beech (Eng.); rooiboekenhout (A); ikhubalo, isifuce (X); umkomiso, uzintlwa (Z) | lanosteryl triterpene | cytotoxicity, antimycobacterial and antimicrobial activities reported | inhibition of ACE | Tree | Bk, L | 1 | [7, 33] |
|  | *Rhus chirindensis* Bak. f. | Red Currant (E)  Bostaaibos (A)  Muvhadela-Phanga (V),  Umhlabamvubu (Z)  Umhlakothi (X) | Phenolics | cytotoxicity, anti-HIV, and antibacterial activities have been reported | Antioxidant and Inhibition of ACE | Tree | L, Rt, Bk, Tw, Fr | 2 | [7, 34, 35] |
|  | *Sclerocarya birrea* A. Rich. | mufula (V); marula (E); maroela (A) | phenolics, saponins, flavonoids, Triterpenes and tannins | antibacterial, antifungal, antihelminthic and antiplasmodial, | exhibiting optimal reduction, and reduced the systemic arterial BP as well as the heart rates | Tree | Sb, L, Fr, Sd | 2 | [36–38] |
|  | *Ozoroa engleri* R. Fern. & A. Fern. | Isifico (Z); mudumbula, bandulakhali (V); dropping resintree (E); treurharpuisboom (A) | NR | Toxicity of the root extract reported | NR | Tree | Rt, Bk, L | 1 | [31, 39] |
|  | *Protorhus longifolia* (Bernh.) Engl. | Uzintlwa (Z); rooimelkhout (A); ikhubalo, isifuce (X) | lanosteryl triterpene | cytotoxicity, antimycobacterial and antimicrobial activities of the crude extract are reported | Inhibition of ACE, inflammation and oxidative stress | Tree | Bk, L | 1 | [23, 33, 40] |
|  | *Searsia burchellii* (Sond. ex Engl.) Moffett (= *Rhus burchellii* Sond. ex Engl. | Karookoeniebos (A) | NR | Antibacterial of leaves and bark have been reported | NR | Shrub/small tree | L, S, Rt | 1 | [18, 41] |
| Apiaceae | *Alepidea amatymbica* | iKhathazo (Z) Iqwili (x), Kalmoes (a), Large Tinsel Flowers | NR | Cytotoxicity, antitrypanosomal, antibacterial, antimicrobial, antifungal have been reported | inhibition of Na+ and K+ reabsorption | Shrub | Rh and Rt | 1 | [42, 43] |
|  | *Lichtensteinia lacera* Cham. & Schltdl. | Kalmiswortel (A) | NR | NR | NR | Herb | L, S | 1 | [18] |
|  | *Peucedanum galbanum* (L.) Drude | Droedas (A) | NR | NR | NR | Shrub | L | 2 | [44] |
| Apocynaceae | *Catharanthus roseus* (L.) G. Dona | musalamarubini (V); Rose periwinkle (E); soldateblom (A) | Alkaloids (Vinblastine and vincristine) | Cytotoxicity as well as anthelmintic, antineoplastic, antidiarrheal, antimicrobial, anticancer have been reported | Inhibition of ACE | Herb | Rt, Fl, Sd | 7 | [31, 39, 45–50] |
|  | *Acokanthera oppositifolia* | inHlungunyembe (Z) | Phenolics | Cytotoxicity reported | Antioxidant activity | Shrub | L, Rt, S | 2 | [25, 51–53] |
|  | *Hoodia gordonii* (Masson) Sweet ex Decne. | Bobbejaanghaap (A) | steroidal glycoside | Non cytotoxic | NR | Herb | L, S | 2 | [18, 27, 54–57] |
|  | *Rauvolfia caffra* sond. | UmHlambamanzi (Z); muṋadzi (V); quinine-tree (E) kinaboom (A) | Alkaloids, alkaloids, terpenoids, flavonoids, polyphenols and saponins | NR | NR | Tree | Bk, S, WP | 2 | [25, 58] |
|  | *Sarcostemma viminale* (L.) R.Br. subsp. suberosum Meve & Liede; | Umbelebele (Z); mutshiso, muṱungu (V); caustic vine (E); spantou, melkbos, melktou (A) | NR | Cytotoxicity reported | NR | Shrub | S, AP, Tw | 3 | [25, 31, 39] |
| Arecaceae | *Hyphaene coriacea* Gaertn. | Ilala (Z); mulala, mutshema (V); lala palm (E); lalapalm (A) | NR | NR | NR | Tree | Rt | 2 | [25, 31, 59] |
|  | *Elaeis guineensis* Jacq | NR | Polyphenolics (catechins) and peptides | Non-cytotoxic (2 – 5 g/kg bw) | endothelium‐dependent mechanism, redox-sensitive phosphorylation of eNOS via the PI3-kinase pathway and ACE inhibition | Tree | Fr, Rt | 4 | [31, 60–65] |
| Asparagaceae | *Drimia elata* Jacq. | Brandui (A); Undongana-zibomvana (Z) | cardiac glycoside, Tannins | Cytotoxicity concern indicated | Antioxidant activity | Herb | B | 2 | [7, 66, 67] |
|  | *Agave americana* L. | Amerikaanse aalwee, kaalgaarboom (A); lekhala (S) | saponin | Cytotoxicity reported | Inhibition of ACE | Shrub | L | 2 | [7, 11, 13, 68] |
| Asphodelaceae | *Aloe marlothii* A.Berger | Inhlaba (Z)  bindamutshe (V); mountain aloe (E); bergaalwyn (A) | NR | Not cytotoxic | NR | Herb | L, Rt | 2 | [25, 39, 69] |
|  | *Aloe ferox* Mill. | iNhlaba (Z) | phenolics, phytosterols, indoles, and alkaloids | Not cytotoxic | antioxidants | Shrub | L/sap | 3 | [18, 26, 60, 70] |
|  | *Aloe striatula* | rankaalwyne (A) | NR | NR | NR | Herb/ Shrub | NR | 1 | [8] |
| Asteraceae | *Senecio serratuloides* DC. | Unsukumbili (Z) | Phenolics | Not cytotoxic | nitric oxide and angiotensin II- like activities |  | L, S | 1 | [39, 71] |
|  | *Schkuhria pinnata* (Lam.) Kuntze ex. Thell | Sebabane (P); Ruhwahwa (Z); Luswielo, gaulo (V); khaki bush (E); khakibos (A) | NR | Not cytotoxic | NR | Herb | WP | 1 | [13, 25] |
|  | *Artemisia afra* Jacq. ex Willd. var. afra | Mhlonyane (Z) Wilde-alsies (A) | NR | Not cytotoxic | NR | Shrub | L | 3 | [18, 26, 60, 72] |
|  | *Chrysocoma ciliata* L. | Bitterbos, Kaalsiektebos (A); bitter cowcud (Eng.); sehalahala (S) | NR | NR | NR | Shrub | L, Rt | 1 | [18] |
|  | *Conyza scabrida* | Umanzimnyama (Z) | NR | NR | NR | Shrub | L | 1 | [18, 26] |
|  | *Dicerothamnus rhinocerotis* (L.f.) Koekemoer | Ranosterbos (A) | methoxylated flavanones | NR | NR | Small shrub | L, S | 1 | [18, 73] |
|  | *Dicoma anomala* Sond. | Umuna (Z); inyongana (X); maagbossie (A) | NR | NR | NR | Herb | L, Rt | 1 | [74] |
|  | *Dicoma capensis* Less. | Koorsbossie (A) | NR | NR | NR | Herb | L, Tw, Rt | 1 | [44] |
|  | *Elytropappus rhinocerotis* (L.f.) Less. | Renosterbos (A) | NR | NR | NR | Tree | Fr, L | 1 | [26] |
|  | *Eriocephalus africanus* | Kapokbos (A) | NR | NR | NR | Shrub | L | 1 | [26] |
|  | *Euryops abrotanifolius* (L.) DC. | Bergharpuisbos, geelmagriet (A) | NR | NR | NR | Shrub | L, S | 1 | [18] |
|  | *Galinsoga parviflora* Cav. | nḓevhedzamphobo (V); yellow weed (E); knopkruid (A) | flavonoids | Antibacterial and anticancer activities reported | Inhibition of ACE and anti-inflammatory | Herb | L | 2 | [5, 75] |
|  | *Helichrysum crispum* (L.) D.Don | Hotnotskooigoed (A) | NR | NR | NR | Herb | L | 1 | [26] |
|  | *Helichrysum odoratissimum* (L.) Sweet. | Imphepho (X and Z); kooigoed, kruie (A) | NR | NR | NR | Herb | L, Rt | 1 | [76] |
|  | *Senecio bupleuroides* DC. | Isiqandamatshana (Z); Idwarane (X), Indabula-luvalo (Z) | NR | NR | NR | Shrub | NR | 1 | [77] |
|  | *Senecio inornatus* DC. | Uhlabo (Z); groot vlei-senecio (A) | NR | NR | NR | Herb/small shrub | Rt | 1 | [77] |
| Balanophoraceae | *Sarcophyte sanguinea* Sparrm. subsp. sanguinea | Umvumbuka (Z); wolwekos (A) | NR | NR | NR | Herb | WP, S, Rt | 1 | [31, 39] |
| Bignoniaceae | *Kigelia africana* (Lam.) Benth. | muvevha (V); sausage-tree (E); worsboom (A) | Flavonoids, phenolics, Iridoids, naphthoquinones, terpenes and phenylethanoglycosides | antimicrobial and anticancer reported | inhibit the oxytocin-induced contraction and anti-inflammatory | Tree | L, Sb, root | 3 | [78–80] |
| Brassiceae | *Cadaba aphylla* (Thunb.) Wild | Swartstormbos, Bobbejaanarm (A)  tshikuni, munnamutswu, mudiatsiwana (V); leafless worm bush (E) | NR | Anticancer activity reported | NR | Shrub | L, St | 2 | [18, 25] |
| Cactaceae | *Opuntia ficus*–indica Mill. | Motloro (P); Umthelekisi (Z); muḓoro (V); sweet prickly pear (E); boereturksvy (A) | Phenolics and flavonoids | Anticancer and antiviral reported | Anti-hyperlipidemic, anti-hypercholesterolemia, anti-inflammatory and antioxidant activities | Shrub/tree | Rt | 3 | [13, 81–83] |
| Canellaceae | *Warburgia ugandensis* | peperbasboom (Afr.); isibhaha (Zulu); manaka (Venda) | terpenoids and polyunsaturated fatty acids | NR | NR | Tree | Sb | 1 | [84, 85] |
|  | *Warburgia salutaris* (G.Bertol.) Chiov. | Isibhaha (Z); mulanga (V); pepper-bark tree (E); peperbasboom (A) | Sesquiterpenoids | NR | NR | Tree | Bk, Rt | 1 | [25, 86, 87] |
| Cannabaceae | *Cannabis sativa* L. | Intsangu (Z); Nsangu (Z) dagga (A); Umya (Xhosa); Matekwane/Patse (S) | flavonoids | Organic extract reported to be mutagenic and cytotoxic | Inhibition of ACE | Herb | L | 5 | [7, 13, 39, 77, 88, 89] |
| Capparaceae | *Cladostemon kirkii* (Oliv.) Pax & Gilg | UmThekwini, isidumbu (Z) | NR | Not cytotoxic | NR | Shrub | Rt, S, Bk | 1 | [31, 39] |
| Celastraceae | *Catha edulis* (Vahl) Forssk. ex Endl. | Umhlwazi (Z); luṱhadzi, mutie (V); bushman-tea, khat (E); boesmanstee (A) | Alkaloids, tannins, flavonoids, terpenes, sterols, and essential oils | Cytotoxicity, genotoxicity, and hepatotoxicity reported. | Inhibition of ACE | Shrub | L | 1 | [7, 25, 39, 77, 90] |
| Commelinaceae | *Commelina africana* L. | Idangabane (Z); lekzotswana (X) | NR | NR | NR | Herb | WP | 4 | [19, 77, 91–94] |
|  | *Commelina benghalensis* L. | Idangabane (Z); damba (V); Benghal commelina (E); Bengaalse wandelende jood (A) | NR | NR | NR | Herb | WP, L | 2 | [8, 25, 77] |
| Convolvulaceae | *Convolvulus capensis* Burm.f. | Skaapklimop (A) | NR | NR | NR | Shrub | B | 1 | [18] |
| Crassulaceae | *Crassula muscosa* L. | Skoenvetebos (A) | NR | NR | NR | Herb | L, S, Rt, Fl | 1 | [18] |
| Cucurbitaceae | *Momordica balsamina* L | Intshungu, Umkaka (Z); tshibavhe, lukake (V); balsam apple (E); aloentjie (A) | resins, alkaloids, flavonoids, glycosides, steroids, terpenes, cardiac glycoside, saponins | antiplasmodial, antimicrobial and antibacterial reported | anti-inflammatory, antioxidant | Herb | L | 2 | [25, 31, 95, 96] |
|  | *Citrullus lanatus* (Thunb.) Matsum. & Nakai | mutshatsha (V); bitter melon (E); bitterappel (A); Ibhece (Z) | Citrulline, cucurbitacins, triterpenes, sterols and alkaloids | Not cytotoxic on RAW264.7 and WRL-68 cells | antioxidant, anti-inflammatory and vasodilatory properties | Herb | Fr, Sd, L | 7 | [31, 39, 97–103] |
|  | *Momordica charantia* L. | lubavhe, tshibavhe (V); balsam pear (E) | Steroidal saponins, peptides, triterpenes, and alkaloids | NR | Reduction of systemic BP and heart rates | Herb | WP | 5 | [96, 104–108] |
|  | *Momordica foetida* Schumach. | Intshungu (Z); nngu (V); gifappel (E); gifappeltjie (A) | Cucurbitane triterpenoids, phenolic and flavonoid | NR | anti-radical activity | Herb | L, S | 3 | [109–111] |
| Ebenaceae | *Euclea undulata* Thunb. | Inkunzane (Z); gewone ghwarrrie (A) | Triterpene | Not cytotoxic to cells | Cardiovascular, vasorelaxant, bradycardia | Shrub | Bk, Rt, WP | 1 | [112–114] |
| Euphorbiaceae | *Ricinus communis* L. var. communis | Umhlakuva (Z); mupfure (V); castor-oil tree (E); kasterolieboom (A) | Tannins, alkaloids, flavonoids | Cytotoxicity of organic extract reported | Anti-inflammatory and free radical scavenging activity | Shrub | L | 1 | [13, 31, 39, 115–117] |
| Fabaceae | *Adenopodia spicata* (E. Mey.) Presl. | umlungumabele (X); ibobo, umbambangwe, (Z) | saponins | NR | Inhibition of ACE | Shrub | L | 1 | [7] |
|  | *Adenopodia spinata* | Ubobo (Z) | NR | NR | NR | Shrub | L, Rt, S | 1 | [7] |
|  | *Arachis hypogaea* L | Amakinati (Z)  nḓuhu, nḓuhumange (V); ground nut (E); grondboontjie (A) | Phenolics, Fatty acids, amino acids and peptides | NR | Inhibition of ACE | Herb | L, Sd | 3 | [118–120] |
|  | *Elephantorrhiza elephantina* (Burch.) Skeels | Intolwane (Z); gumululo, gumbathakha (V); sumach bean, dwarf elephant-root (E); elandsboontjie (A) | epicatechin and palmitic acid | Antibacterial and cytotoxicity reported | Antioxidant | Sub-shrub | L, Rh | 3 | [77, 121–123] |
|  | *Lessertia frutescens* (L.) Goldblatt & J.C.Manning subsp. frutescens (= *Sutherlandia frutescens* (L.) R.Br.) | Umnwele (X and Z); klappers, hoenderbelletjie (A) | Saponins, alkaloids, flavonoids | NR | Antioxidant | Shrub | L | 2 | [8, 18, 124] |
|  | *Medicago sativa* L. | Klawer (A) | Saponins, phenolics | NR | Inhibition of ACE | Herb | WP | 4 | [13, 125–128] |
|  | *Tephrosia capensis* | Fish Bean (E) | Flavonoids, alkaloids | NR | NR | Small shrub | Rt | 1 | [74, 123, 129] |
|  | *Trifolium africanum* Ser. | Wildeklawer (A); moqophi, (S) | NR | NR | NR | Herb | WP | 2 | [19, 74, 130] |
|  | *Trifolium burchellianum* Benth N.E.Br. | Usithathi (Z) | NR | NR | NR | Herb | L, S, Rt | 2 | [19, 123, 131] |
| Geraniaceae | *Geranium incanum* Burm.f. | Vrouetee (A); ngope-sethsoha, tlako (Sotho) | Tannins, flavonoids | Not toxic in mice | NR | Small herb | L, S | 1 | [89, 132] |
| Hyacinthaceae | *Drimia robusta* Bak. | Brandui (A), Isiklenama (Z) | alkaloids, saponins and cardiac glycosides | Antibacterial activity reported | Inhibition of ACE |  | L, B, Rt | 3 | [7, 133–135] |
|  | *Dipcadi brevifolium* | Ikhakahkha (Z); Slangui (A) | NR | NR | NR | Herb | B | 1 | [77] |
| Hypoxidaceae | *Hypoxis argentea*  Harv. ex Baker | Labateka, Inongwe (Z) | glucosides, sterols and sterolins | NR | NR | Herb | C, T | 2 | [31, 136, 137] |
|  | *Hypoxis colchicifolia* Bak. | broad-leaved hypoxis (E); iLabatheka (Z) | pentacyclic tritepenoid ursolic acid | Antibacterial activities reported | Antioxidant and anti-inflammatory activity | Herb | B | 2 | [7, 138, 139] |
|  | *Hypoxis hemerocallidea* Fisch., C.A.Mey. & Avé-Lall. | Inkomfe (Z); ikhubalo lezithunzela (X) | phytosterol glucosides, aglycone rooperol, sterols, and sterolins | Not cytotoxic |  | Small herb | C, Rt | 6 | [25, 39, 89, 123, 131, 140] |
| Icacinaceae | *Pyrenacantha kaurabassana* Baill. | Inseam (Z); galange (V) | NR | Roots cytotoxic in BSLA | NR | Shrub | f | 1 | [31, 141, 142] |
| Iridaceae | Dietes iridioides (L.) Sweet ex Klatt | wilde-iris (A); indawo-yehlathi, isishuphe somfula (Z) | Flavonoids | NR | the lower left ventricular systolic pressure, aortic blood pressure through the decrease in dP/dtmax and inhibition of ACE | Herb | L, Rt, Rh | 3 | [6, 77, 143, 144] |
| Lamiaceae | *Tetradenia riparia* (Hochst.) Codd | Ibozane (Z); watersalie (A) | NR | NR | NR | Shrub/tree | L,Sd, Rt | 4 | [18, 31, 39, 145, 146] |
|  | *Ballota africana (L.) Benth.* | Kattekruide (A) | NR | NR | Inhibition of ACE | Shrub | L | 5 | [7, 18, 26, 74, 146] |
|  | *Leonotis leonurus* (L.) R. Br. | Umfincafincane (Z); muṋunzu (V); wilde dagga, narrow-leaved leonotis (E); wildedagga (A) | Terpenoids, alkaloid, diterpene | NR | β1  agonist effect and direct vasoconstrictive effect | Shrub | L, Rt, Fl, WP | 5 | [18, 25, 71, 131, 147–149] |
|  | *Mentha aquatica* L. | Amabunu (Z), Ityaleba (X) | Phenolics, fatty acids, amino acids, pentacyclic triterpenes | NR | NR | Herb | L, S, Sd | 2 | [19, 74, 131, 150] |
|  | *Mentha longifolia* L. | ufuthana lomhlanaga (Z); inzinziniba (X) | NR | Not toxic in rats | NR | Herb | L, S | 2 | [18, 151, 152] |
|  | *Ocimum basilicum* L. | Sweet basil (E) | Phenolics and flavonoids | Not cytotoxic | Inhibition of ACE and endothelium-dependent vasorelaxant | Herb | L, S | 3 | [153–155] |
|  | *Salvia africana* L. (= *Salvia africana-caerulea* L.) | Wildesalie (A) | NR | NR | NR | Shrub | Tw, L | 1 | [18] |
| Lauraceae | *Persea americana* Mill. | Moafokhath (P); mugadaphele (V); avocado (E); avokado (A) | Alkaloids, steroids, flavonoids and saponins | Not toxic | Antioxidant and vasorelaxation | Tree | L, P, Fr, Rt | 2 | [30, 156, 157] |
|  | *Cinnamomum camphora* L. | Uroselina (Z) | Rutin and quercetin | NR | Amelioration of oxidative stress and anti-inflammatory activity | Tree | Gum, Sd | 2 | [158–161] |
| Lythraceae | *Punica granatum* L. | Mokgarenat (P) | polyphenolics, tannins and anthocynins | antibacterial reported | inflammatory activity, inhibition of ACE and amelioration of oxidative stress | Shrub | Rt | 2 | [162–164] |
| Malvaceae | *Dombeya rotundifolia* (Hochst.) Planch var. rotundifolia | tshiluvhari (V); wild-pear (E); wildepeer, drolpeer (A); INhliziyonkhulu (Z); drolpeer (A); nsihaphukuma (T) | saponins, tannins, and cardiac glycosides | antibacterial activity reported | anti-inflammatory and inhibition of ACE | Tree | L, Rt, S | 3 | [165–167] |
| Meliaceae | *Trichilia emetica* Vahl | Umkhuhlu (Z), mutshikili (V); Natal-mahogany (E); rooiessenhout (A) | Flavonoids, Limonoids, Tannins, trichilin A, rohituka 3, seco-A-protolimonoid | Slightly toxic | Antioxidant, anti-inflammatory | Tree | L, Fr, Rt, Bk, S | 5 | [25, 31, 77, 131, 168–170] |
|  | *Ekebergia capensis* Sparrm | muṱobvuma, muzhouzhou (V); Cape-ash (E); essenhout (A) | Triterpenes, saponins, glycosides, polyphenols, and tannins | Cytotoxicity reported | EDRF-dependent or -independent pathways | Tree | L, Bk | 4 | [7, 171–174] |
|  | *Turraea floribunda* Hochst. | Umadlozane (Z); kanferfoelieboom (A); wild honeysuckle-tree (Eng.) | Flavonoids, phenolics, limonoids, turraflorins, tetranortriterpenoids | Cytotoxicity reported | antioxidant, anti-inflammatory activities, and inhibition of ACE | Tree | Bk, L, Rt | 2 | [7, 175] |
| Menispermaceae | *Albertisia delagoensis* (N.E.Br.) Forman | Ohumane, Umgandaganda (Z) | Alkaloids | Organic root extract cytotoxicity reported | NR | Shrub | Rh, L, S, Rt | 3 | [31, 39, 176, 177] |
|  | *Cissampelos capensis* L.f. | Dawidjieswortel, Fynblaarklimop (A); mayisake (X) | Alkaloids | Contains toxic alkaloids | Anti-inflammatory activity | Shrub | Rt | 4 | [89, 131, 178–180] |
| Mesembry-  anthemaceae | *Carpobrotus dimidiatus* (Haw.) L.Bolus | Ikhambi lamabulawo (Z); Natalse suurvy/strandvy (A) | NR | Not cytotoxic at 1000 μg/ml in BSLA | Inhibition of ACE and anti-inflammatory activity | Herb | L, S, Fr | 2 | [31, 39] |
| Musaceae | *Musa acuminata* Colla | ihliziyo kabhanana ebomvu (Z); muombva (V); banana; piesangboom (A) | Phenolics, saponins, terpenoids, steroids, anthocyanins, fatty acids, tannins, and alkaloids | Not cytotoxic | NR | Herb | FL | 6 | [25, 31, 39, 181–184] |
| Myrothamnaceae | *Myrothamnus flabellifolius* Welw. | mafautshivuwa, mafavuka (V); resurrection plant (E); bergboegoe (A) | Phenolics, alkaloids, flavonoids, glycosides, saponin, tannin, phytosterol, and triterpene | NR | Antioxidant | Shrub | L, Tw | 3 | [25, 142, 185–187] |
| Myrsinaceae | *Rapanea melanophloeos* L. | IKhubalwane (Z); tshiḓiḓiri (V); Cape-beech (E); Kaapse boekenhout (A) | Saponins, triterpenoids | NR | Antioxidant | Tree | Bk | 2 | [25, 77, 131, 142, 188] |
| Myrtaceae | *Psidium guajava* L. | Ugwava (Z); Koejawel (A); Mokwaba (P); mugwavha (V); guava (E) | Phenolic (Rosmarinic acid, eugenol, carvacrol, catechin and caffeic acid) and flavonoids | Not toxic, antigenotoxic, antimutagenic and antimicrobial activities reported | Inhibition of ACE and antioxidant activity | Shrub/small tree | L, Rt | 10 | [31, 39, 142, 189–199] |
| Oleaceae | *Olea europaea* subsp. africana | Umnquma (Z); muṱwari (V); wild olive (E); olienhout (A) | Phenolic, flavonoids, essential oils, glycosides, sugars, sterols, triterpenoids, and secoiridoids | Anticancer and antimicrobial activities reported | Inhibition of ACE | Tree | L, Rt | 4 | [5, 173, 200–203] |
| Polygonaceae | *Oxygonum sinuatum* (Hochst. & Steud. ex Meisn.) | Unknown | diterpenoid | NR | Inhibition of ACE | Herb | L | 2 | [5, 204] |
| Pteridaceae | *Adiatum capillus*-veneris L. | NK | tannins, terpenoids, flavonoids, alkaloids, and steroids | NR | Lowering of systolic blood  pressure | shrub | L | 3 | [7, 205–207] |
| Rosaceae | *Eriobotrya japonica* (Thunb.) Lindl. | munombelo (V); loquat (E); lukwart (A) | triterpene | NR | cardio-protective and anti-inflammatory effects | Shrub | L, Fr | 1 | [13, 208, 209] |
|  | *Leucosidea sericea* Eckl. & Zeyh. | Umtshitshi (Z); ouhout (A); umyityi (X) | phenolics | NR | Anti-inflammatory activity | Small tree | NR | 1 | [74, 210] |
| Rubiaceae | *Vangueria infausta* Burch. subsp. infausta | Umvilo, umTulwa (Z); muzwilu (V); velvet wild-medlar (E); grootmispel, wildemispel (A) | triterpenoid | Not cytotoxic | NR | Shrub/small tree | Bk, L | 2 | [31, 39, 142, 211] |
|  | *Oldenlandia affinis* (Roem. & Schult.) DC. subsp. | Umampeshane (Z) | peptide | NR | NR | Herb | Rt | 1 | [77] |
|  | *Pentanisia prunelloides* (Klotzsch ex Eckl. & Zeyh.) Walp. | Icimamlilo (Z); sooibrandbossie (A) | tannins, terpenoids, alkaloids, saponins, flavonoids and cardiac glycosides | NR | Anti-inflammatory and antioxidant activities | Herb | Rh, C, Rt | 1 | [77, 122, 212, 213] |
|  | *Spermacoce natalensis* Hochst. | Umabophe (Z) | NR | NR | NR | Herb | Bk, Rt, L | 1 | [77] |
| Rutaceae | *Citrus limon* (L.) Osbecka | Ulamula (Z); tshikavhavhe, mukavhavhe (V); lemon (E); suurlemoen (A) | phenolic acids, flavonoids, triterpenoids, carotenoids, essential oils, and tannins | NR | Antioxidant and anti-inflammatory activities | Shrub | Fr peel, P, Rt | 4 | [31, 214–217] |
|  | *Citrus maxima* (Burm.) Merr.a | Upapamuzi (Z) | Phenolics, flavonoid, Monoterpenes, Sesquiterpenes | NR | Antioxidant, anti-inflammatory activities and Inhibition of ACE | Tree | Fr | 3 | [215, 218, 219] |
|  | *Toddalia asiatica* (L.) Lam. | gwambadzi, mufhiso, tshikwangula (V); climbing-orange (E); ranklemoentjie (A) | Alkaloids, coumarins, quinoline | Cytotoxicity reported in cancer cells | Anti-inflammatory and antioxidant activity | Tree | L, Rt | 1 | [85, 220–222] |
|  | *Agathosma betulina* | Regteboegoe (A) | limonene, menthone, diosphenol, l-pulegone and (ψ)-diosphenol | Not cytotoxic at 100 µg/ml | Antioxidant and anti-inflammatory activity | Shrub | L, S | 2 | [89, 223, 224] |
|  | *Clausena anisata* (Willd.) Hook.f. ex Benth. | Umnukambhiba (Z); Isifutho (X); Perdepisbossie (A) | phenolics,terpenoids, sesquiterpenes, fatty acids, alkaloids, coumarins, limonoids, tannins, and saponins |  | Inhibition of ACE | Shrub | L, Rt | 6 | [7, 77, 225–228] |
|  | *Diosma oppositifolia* L. | Bitterboegoe (A) | NR | NR | NR | Shrub | L, S, Fl | 1 | [18] |
|  | *Ptaeroxylon obliquum* (Thunb.) Radlk. | UmThathi (X); Umsango (Z) | prenylated coumarins and chromones | Organic extract showed cytotoxicity | Antioxidant and anti-inflammatory activities | Tree | Rt | 4 | [18, 31, 39, 229, 230] |
|  | *Ruta graveolens* | Binnewortel (A) | acridone alkaloids, coumarins, essential oils, flavonoids and furoquinolines | NR | Reduction of oxidative stress, inflammation and aortic pathology. vasorelaxant via the nitric oxide-guanylyl cyclase pathway and a prostaglandin-mediated mechanism, as well as activation of the ATP-sensitive potassium channel | Shrub | L | 6 | [26, 44, 89, 142, 231–235] |
| Salicaceae | *Scolopia mundii* (Eckl. & Zeyh.) Warb. | iHlambahlale (Z); klipdoring (A) | NR | NR | NR | Tree | Bk | 1 | [77] |
| Scrophulariaceae | *Teedia lucida* (Sol.) Rudolphi | Hlwenya (Z); Klipkersie (A) | NR | NR | NR | Herb/dwarf shrub | Rt | 2 | [74, 230] |
| Solanaceae | *Physalis viscosa* L. | NR | NR | NR | NR | Herb | L | 2 | [5, 6] |
| Strychnaceae | *Strychnos madagascariensis* Poir. | Umkwakwa (Z); mukwakwa (V); black monkey-orange (E); swartklapper (A) | Anthraquinones, flavonoids, tannins, and saponins | Poisonous in fish | NR | Tree | Sd, Bk, Rt, Fr | 1 | [31, 39, 60] |
| Urticaceae | *Urtica urens* L. | Imbabazane (X), Imbathi (Z), Perdebrandnekel (A) | Phenolics, saponin | Not toxic | Anti-inflammatory | Herb | L | 2 | [19, 236–239] |
| Valerianaceae | *Valeriana capensis* var. | Wildebalderjan (A) | NR | NR | Inhibition of ACE | Herb | Rh, Rt |  | [44, 240] |
| Verbanaceae | *Lippia javanica* (Burm.f.) Spreng | Umsuzwane (Z); inzinziniba (X); lemoenbossie (A) | Phenolics, flavonoid, amino acids, essential oil, sesquiterpenoids, triterpenoids and iridoid glycosides | Triterpenoids reported toxic | NR | Shrub | L | 2 | [31, 241–243] |
| Verbenaceae | *Lantana camara* L. | ebabane/motsholla (P); Ubukhwebezane (Z); tshidzimbampoṱolo (V); cherry-pie, common lantana (E); boesmandruiwe (A) | Flavonoids, triterpenes, Phenylpropanoid Glycosides, Ursolic and oleanolic acids | Cytotoxicity of some compounds reported | Prevention of oxidative stress | Shrub | Rt | 5 | [13, 244–248] |
| Vitaceae | *Rhoicissus digitata* (L.f.) Gilg & M.Brandt | UmNangwazi (Z); bobbejaandruif (A); isaQoni (X) | phenolics | Anticandidal and antifungal activities reported | Anti-inflammation and antioxidant activities | Shrub | T, B | 2 | [89, 131, 142, 249–252] |
| Zamiaceae | Stangeria eriopus (Kunze) Baill. | Natal grass cycad (E); bobbejaankos (A); Imfingo (Z); Umfigwani (X); bobbejaankos (A) | Glycosides, biflavones | NR | Inhibition of ACE | Cycad | Rt, L | 2 | [7, 77, 253, 254] |

Part used: Bk: Bark; Rh: Rhizome L: leaves; Rb, root bark; Sb: stem bark; WP: whole plant; B: Bulbs; Ap: Aerial part; Fr: Fruit; Hp: Herbal parts; T: Tuber; F: Flower; S: stem; Ep: external part; Rt: roots; Sd: seeds; Tw: Twigs. NR: Not reported

Vernacular names: Z: Zulu; X: isiXhosa; V: Venda; P: Pedi; A: Africans; T: Tsonga; S: Sesotho

**References**

[1] P. R, S. Dodoala, M. S. Sheikuduman, C. Chetty, and R. D, “Potential hypoglycemic & hypolipidemic effect of Morus Indica and Asystasia gangetica in alloxan induced diabetes mellitus,” *Int. J. Res. Pharm. Sci.*, vol. 1, 2010.

[2] K. Sama, R. Sivaraj, H. Abdul Salam, and R. Raju, “Pharmacognostical and Phytochemical Screening of Asystasia gangetica (Chinese Violet),” *Int. Res. J. Pharm.*, vol. 4, pp. 161–163, 2013.

[3] N. Tsabang, C. G. Yedjou, L. Tsambang, et al., “Treatment of Diabetes and/or Hypertension Using Medicinal Plants in Cameroon.,” *Med. Aromat. plants*, vol. Suppl 2, 2015.

[4] P. Mugabo and I. A. Raji, “Effects of aqueous leaf extract of Asystasia gangetica on the blood pressure and heart rate in male spontaneously hypertensive Wistar rats,” *BMC Complement. Altern. Med.*, vol. 13, no. 1, p. 283, Dec. 2013.

[5] S. Ramesar, H. Baijnath, T. Govender, and I. Mackraj, “Angiotensin I-Converting Enzyme Inhibitor Activity of Nutritive Plants in KwaZulu-Natal,” *J. Med. Food*, vol. 11, no. 2, pp. 331–336, Jun. 2008.

[6] P. De Lange-Jacobs, A. Shaikh-Kader, B. Thomas, and T. T. Nyakudya, “An Overview of the Potential Use of Ethno-Medicinal Plants Targeting the Renin–Angiotensin System in the Treatment of Hypertension,” *Molecules*, vol. 25, no. 9, p. 2114, Apr. 2020.

[7] A. C. Duncan, A. K. Jäger, and J. van Staden, “Screening of Zulu medicinal plants for angiotensin converting enzyme (ACE) inhibitors,” *J. Ethnopharmacol.*, vol. 68, no. 1–3, pp. 63–70, Dec. 1999.

[8] F. Balogun and A. Ashafa, “A Review of Plants Used in South African Traditional Medicine for the Management and Treatment of Hypertension,” *Planta Med.*, vol. 85, no. 04, pp. 312–334, Mar. 2019.

[9] N. F. Mohamed Yahaya, M. A. Rahman, and N. Abdullah, “Therapeutic potential of mushrooms in preventing and ameliorating hypertension,” *Trends Food Sci. Technol.*, vol. 39, no. 2, pp. 104–115, Oct. 2014.

[10] C. C. Lau, N. Abdullah, A. S. Shuib, and N. Aminudin, “Novel angiotensin I-converting enzyme inhibitory peptides derived from edible mushroom Agaricus bisporus (J.E. Lange) Imbach identified by LC–MS/MS,” *Food Chem.*, vol. 148, pp. 396–401, Apr. 2014.

[11] N. Monterrosas-Brisson, M. Ocampo, E. Jiménez-Ferrer, et al., “Anti-Inflammatory Activity of Different Agave Plants and the Compound Cantalasaponin-1,” *Molecules*, vol. 18, no. 7, pp. 8136–8146, Jul. 2013.

[12] S. G. Sparg, M. E. Light, and J. van Staden, “Biological activities and distribution of plant saponins,” *J. Ethnopharmacol.*, vol. 94, no. 2–3, pp. 219–243, Oct. 2004.

[13] S. Semenya, M. Potgieter, M. Tshisikhawe, S. Shava, and A. Maroyi, “Medicinal utilization of exotic plants by Bapedi traditional healers to treat human ailments in Limpopo province, South Africa,” *J. Ethnopharmacol.*, vol. 144, no. 3, pp. 646–655, Dec. 2012.

[14] S. M. S. J. S. Kang, “Inhibitory Effect of Cell Differentiation against 3T3-L1 Pre-Adipocytes and Angiotensin Converting Enzyme (ACE) Activity of Ice Plant (Mesembryanthemum crystallinum),” *J. Korean Soc. Food Sci. Nutr.*, vol. 46, no. 8, pp. 1012–1017, 2017.

[15] K. Sakamoto and K. Megumi, “Mesembryanthemum crystallinum extract suppressed the early differentiation of mouse 3T3-L1 preadipocytes,” *J. Nat. Pharm.*, vol. 2, no. 4, p. 184, 2011.

[16] C. Cosentino, “Na+/H+ transporters of the halophyte Mesembryanthemum crystallinum L.,” 2008.

[17] H. Nell, M. Siebert, P. Chellan, and N. Gericke, “A Randomized, Double-Blind, Parallel-Group, Placebo-Controlled Trial of Extract *Sceletium tortuosum* (Zembrin) in Healthy Adults,” *J. Altern. Complement. Med.*, vol. 19, no. 11, pp. 898–904, Nov. 2013.

[18] D. Davids, D. Gibson, and Q. Johnson, “Ethnobotanical survey of medicinal plants used to manage High Blood Pressure and Type 2 Diabetes Mellitus in Bitterfontein, Western Cape Province, South Africa,” *J. Ethnopharmacol.*, vol. 194, pp. 755–766, Dec. 2016.

[19] A. Moteetee, “The medical ethnobotany of Lesotho,” *Bothalia -Pretoria-*, vol. 41, pp. 209–228, 2011.

[20] K. Moodley, I. Mackraj, and Y. Naidoo, “Cardiovascular effects of Tulbaghia violacea Harv. (Alliaceae) root methanolic extract in Dahl salt-sensitive (DSS) rats,” *J. Ethnopharmacol.*, vol. 146, no. 1, pp. 225–231, Mar. 2013.

[21] I. Raji, K. Obikeze, and P. Mugabo, “Potential Beneficial Effects of&lt;i&gt; Tulbaghia violacea&lt;/i&gt; William Henry Harvey (Alliaceae) on Cardiovascular System - A Review,” *Trop. J. Pharm. Res.*, vol. 14, no. 6, p. 1111, Jul. 2015.

[22] A. O. Aremu and J. Van Staden, “The genus Tulbaghia (Alliaceae)—A review of its ethnobotany, pharmacology, phytochemistry and conservation needs,” *J. Ethnopharmacol.*, vol. 149, no. 2, pp. 387–400, Sep. 2013.

[23] J. S. Miller, “Zulu Medicinal Plants:  An Inventory By A. Hutchings with A. H. Scott, G. Lewis, and A. B. Cunningham (University of Zululand). University of Natal Press, Pietermaritzburg. 1996. xiv + 450 pp. 21 × 29.5 cm. $133.00. ISBN 0-86980-893-1.,” 1997.

[24] U. A. Bhosale, R. Yegnanarayan, P. Pophale, and R. Somani, “Effect of aqueous extracts of Achyranthes aspera Linn. on experimental animal model for inflammation.,” *Anc. Sci. Life*, vol. 31, no. 4, pp. 202–6, Apr. 2012.

[25] K. Magwede, B.-E. van Wyk, and A. E. van Wyk, “An inventory of Vhavenḓa useful plants,” *South African J. Bot.*, vol. 122, pp. 57–89, May 2019.

[26] T. S. A. S. A. Thring and F. M. M. Weitz, “Medicinal plant use in the Bredasdorp/Elim region of the Southern Overberg in the Western Cape Province of South Africa.,” *J. Ethnopharmacol.*, vol. 103, no. 2, pp. 261–75, Jan. 2006.

[27] T. T. Nyakudya, T. Tshabalala, R. Dangarembizi, K. H. Erlwanger, and A. R. Ndhlala, “The Potential Therapeutic Value of Medicinal Plants in the Management of Metabolic Disorders,” *Molecules*, vol. 25, no. 11, p. 2669, Jun. 2020.

[28] P. Mugabo, K. C. Obikeze, A. Njagi, and A. P. Burger, “Cardiovascular effects of the alkaloid hippadine on the isolated perfused rat heart,” *Int. J. Med. Aromat. Plants*, vol. 2, pp. 172–177, 2012.

[29] P. Mugabo, A. Philander, I. Raji, D. Dietrich, and I. Green, “Effects of hippadine on the blood pressure and heart rate in male spontaneously hypertensive Wistar rats,” *J. Ethnopharmacol.*, vol. 158, pp. 123–131, Dec. 2014.

[30] S. Semenya, M. Potgieter, and L. Erasmus, “Ethnobotanical survey of medicinal plants used by Bapedi healers to treat diabetes mellitus in the Limpopo Province, South Africa,” *J. Ethnopharmacol.*, vol. 141, no. 1, pp. 440–445, May 2012.

[31] H. de Wet, M. Ramulondi, and Z. N. Ngcobo, “The use of indigenous medicine for the treatment of hypertension by a rural community in northern Maputaland, South Africa,” *South African J. Bot.*, vol. 103, pp. 78–88, Mar. 2016.

[32] S. Nciki, S. Vuuren, A. van Eyk, and H. de Wet, “Plants used to treat skin diseases in northern Maputaland, South Africa: antimicrobial activity and *in vitro* permeability studies,” *Pharm. Biol.*, vol. 54, no. 11, pp. 2420–2436, Nov. 2016.

[33] R. Mosa, G. Lazarus, Gwala, A. Oyedeji, and N. Opoku, “In Vitro Anti-platelet Aggregation, Antioxidant and Cytotoxic Activity of Extracts of Some Zulu Medicinal Plants,” *J. Nat. Prod.*, vol. 4, pp. 136–146, 2011.

[34] J. A. O. Ojewole, “Analgesic, anti-inflammatory and hypoglycaemic effects of Rhus chirindensis (Baker F.) [Anacardiaceae] stem-bark aqueous extract in mice and rats,” *J. Ethnopharmacol.*, vol. 113, no. 2, pp. 338–345, Sep. 2007.

[35] D. I. Viol, L. S. Chagonda, T. Munodawafa, et al., “Antioxidant Activity and Total Phenolic Contents of some Traditional Medicinal Plants from Zimbabwe,” *J. Biol. Act. Prod. from Nat.*, vol. 3, no. 5–6, pp. 345–352, Nov. 2013.

[36] † Alessandra Braca, † Matteo Politi, # Rokia Sanogo, et al., “Chemical Composition and Antioxidant Activity of Phenolic Compounds from Wild and Cultivated Sclerocarya birrea (Anacardiaceae) Leaves,” 2003.

[37] J. A. O. Ojewole, “Vasorelaxant and hypotensive effects of Sclerocarya birrea (A Rich) Hochst (Anacardiaceae) stem bark aqueous extract in rats.,” *Cardiovasc. J. South Africa*, vol. 17, no. 3, pp. 117–123, 2006.

[38] J. A. O. Ojewole, T. Mawoza, W. D. H. Chiwororo, and P. M. O. Owira, “Sclerocarya birrea (A. Rich) Hochst. [’Marula’] (Anacardiaceae): a review of its phytochemistry, pharmacology and toxicology and its ethnomedicinal uses.,” *Phytother. Res.*, vol. 24, no. 5, pp. 633–9, May 2010.

[39] M. Ramulondi, H. de Wet, and S. van Vuuren, “Toxicology of medicinal plants and combinations used in rural northern KwaZulu-Natal (South Africa) for the treatment of hypertension,” *J. Herb. Med.*, vol. 16, p. 100251, Jun. 2019.

[40] S. E. Mabhida, R. Johnson, M. Ndlovu, et al., “A Lanosteryl triterpene from Protorhus longifolia augments insulin signaling in type 1 diabetic rats,” *BMC Complement. Altern. Med.*, vol. 18, no. 1, p. 265, Dec. 2018.

[41] T. R. H. Nielsen, V. Kuete, A. K. Jäger, J. J. M. Meyer, and N. Lall, “Antimicrobial activity of selected South African medicinal plants.,” *BMC Complement. Altern. Med.*, vol. 12, p. 74, Jun. 2012.

[42] O. A. Wintola and A. J. Afolayan, “*Alepidea amatymbica* Eckl. &amp; Zeyh.: A Review of Its Traditional Uses, Phytochemistry, Pharmacology, and Toxicology,” *Evidence-Based Complement. Altern. Med.*, vol. 2014, pp. 1–11, 2014.

[43] L. . Somova, F. . Shode, K. Moodley, and Y. Govender, “Cardiovascular and diuretic activity of kaurene derivatives of Xylopia aethiopica and Alepidea amatymbica,” *J. Ethnopharmacol.*, vol. 77, no. 2–3, pp. 165–174, Oct. 2001.

[44] B. . Van Wyk, B. Van Oudtshoorn, and N. Gericke, *Medicinal Plants of South Africa*, Reprint. Pretoria: Briza Publications, Pretoria, 2002.

[45] N. Ara, M. Rashid, and M. Amran, “Comparison of hypotensive and hypolipidemic effects of Catharanthus roseus leaves extract with atenolol on adrenaline induced hypertensive rats,” *Pak. J. Pharm. Sci.*, vol. 22, pp. 267–271, 2009.

[46] M. Mathiu, “Acute toxicity studies of Catharanthus roseus aqueous extract in male Wistar rats,” *African J. Pharmacol. Ther.*, pp. 130–134, 2015.

[47] D. M. Pereira, F. Ferreres, J. M. A. Oliveira, et al., “Pharmacological effects of Catharanthus roseus root alkaloids in acetylcholinesterase inhibition and cholinergic neurotransmission,” *Phytomedicine*, vol. 17, no. 8–9, pp. 646–652, Jul. 2010.

[48] E. Noumi, F. Houngue, and D. Lontsi, “Traditional medicines in primary health care: plants used for the treatment of hypertension in Bafia, Cameroon,” *Fitoterapia*, vol. 70, no. 2, pp. 134–139, Apr. 1999.

[49] L. Kevin, A. Hussin, I. Zhari, and J. Chin, “Sub–acute oral toxicity study of methanol leaves extract of Catharanthus roseus in rats,” *J. Acute Dis.*, vol. 1, no. 1, pp. 38–41, 2012.

[50] A. Nisar, A. Mamat, M. I. H. Mohamed Dzahir, M. S. Ahmad, and M. Aslam, “An Updated Review On Catharanthus Roseus: Phytochemical And Pharmacological Analysis,” *Indian Res. J. Pharm. Sci.*, vol. 3, pp. 631–653, 2016.

[51] M. E. M. Saeed, M. Meyer, A. Hussein, and T. Efferth, “Cytotoxicity of South-African medicinal plants towards sensitive and multidrug-resistant cancer cells,” *J. Ethnopharmacol.*, vol. 186, pp. 209–223, Jun. 2016.

[52] A. A. Adedapo, F. O. Jimoh, A. J. Afolayan, and P. J. Masika, “Antioxidant activities and phenolic contents of the methanol extracts of the stems of Acokanthera oppositifolia and Adenia gummifera,” *BMC Complement. Altern. Med.*, vol. 8, no. 1, p. 54, Dec. 2008.

[53] S. A. Adebayo, M. Ondua, L. J. Shai, and S. L. Lebelo, “Inhibition of nitric oxide production and free radical scavenging activities of four South African medicinal plants.,” *J. Inflamm. Res.*, vol. 12, pp. 195–203, 2019.

[54] O. Roza, N. Lovász, I. Zupkó, J. Hohmann, and D. Csupor, “Sympathomimetic Activity of a *Hoodia gordonii* Product: A Possible Mechanism of Cardiovascular Side Effects,” *Biomed Res. Int.*, vol. 2013, pp. 1–6, 2013.

[55] V. Madgula, B. Avula, R. Pawar, et al., “*In Vitro* Metabolic Stability and Intestinal Transport of P57AS3 (P57) from *Hoodia gordonii* and its Interaction with Drug Metabolizing Enzymes,” *Planta Med.*, vol. 74, no. 10, pp. 1269–1275, Aug. 2008.

[56] I. Vermaak, J. Hamman, and A. Viljoen, “*Hoodia gordonii:* An Up-to-Date Review of a Commercially Important Anti-Obesity Plant,” *Planta Med.*, vol. 77, no. 11, pp. 1149–1160, Jul. 2011.

[57] P. Kapewangolo, M. Knott, R. E. K. Shithigona, S. L. Uusiku, and M. Kandawa-Schulz, “In vitro anti-HIV and antioxidant activity of Hoodia gordonii (Apocynaceae), a commercial plant product,” *BMC Complement. Altern. Med.*, vol. 16, no. 1, p. 411, Dec. 2016.

[58] T. K. Milugo, L. K. Omosa, J. O. Ochanda, et al., “Antagonistic effect of alkaloids and saponins on bioactivity in the quinine tree (Rauvolfia caffra sond.): further evidence to support biotechnology in traditional medicinal plants.,” *BMC Complement. Altern. Med.*, vol. 13, p. 285, Oct. 2013.

[59] M. Gruca, T. R. van Andel, and H. Balslev, “Ritual uses of palms in traditional medicine in sub-Saharan Africa: a review,” *J. Ethnobiol. Ethnomed.*, vol. 10, no. 1, p. 60, Jul. 2014.

[60] N. C. Dlova and M. A. Ollengo, “Traditional and ethnobotanical dermatology practices in Africa,” *Clin. Dermatol.*, vol. 36, no. 3, pp. 353–362, May 2018.

[61] J. M. Jaffri, S. Mohamed, N. Rohimi, et al., “Antihypertensive and Cardiovascular Effects of Catechin-Rich Oil Palm ( *Elaeis guineensis* ) Leaf Extract in Nitric Oxide–Deficient Rats,” *J. Med. Food*, vol. 14, no. 7–8, pp. 775–783, Jul. 2011.

[62] V. U. Anyanji, S. Mohamed, and H. Bin Bejo, “Acute toxicity and safety assessment of oil palm (Elaeis guineensis Jacq.) leaf extract in rats,” *J. Med. Plants Res.*, vol. 7, no. 16, pp. 1022–1029, Apr. 2013.

[63] M. Abeywardena, I. Runnie, M. Nizar, R. Head, and R. Head, “Polyphenol-enriched extract of oil palm fronds (Elaeis guineensis) promotes vascular relaxation via endothelium-dependent mechanisms,” *Asia Pac. J. Clin. Nutr.*, vol. 11, no. s7, pp. S467–S472, Oct. 2002.

[64] Y. Zheng, Y. Li, Y. Zhang, X. Ruan, and R. Zhang, “Purification, characterization, synthesis, in vitro ACE inhibition and in vivo antihypertensive activity of bioactive peptides derived from oil palm kernel glutelin-2 hydrolysates,” *J. Funct. Foods*, vol. 28, pp. 48–58, Jan. 2017.

[65] M. Ndiaye, E. Anselm, M. Séne, et al., “Mechanisms underlying the endothelium-dependent vasodilatory effect of an aqueous extract of Elaeis guineensis Jacq. (Arecaceae) in porcine coronary artery rings,” *African J. Tradit. Complement. Altern. Med.*, vol. 7, no. 2, Feb. 2010.

[66] M. Bozorgi, G. Amin, M. Shekarchi, and R. Rahimi, “Traditional medical uses of Drimia species in terms of phytochemistry, pharmacology and toxicology,” *J. Tradit. Chinese Med.*, vol. 37, no. 1, pp. 124–139, Feb. 2017.

[67] A. Maroyi, “Review Of Medicinal Uses, Phytochemistry, And Pharmacological Properties Of Drimia elata,” *Asian J. Pharm. Clin. Res.*, pp. 37–44, Mar. 2019.

[68] B. A. A. Mannasaheb, P. V Kulkarni, M. A. Sangreskopp, C. Savant, and A. Mohan, “Protective effect of Agave americana Linn. leaf extract in acetic acid-induced ulcerative colitis in rats.,” *Ayu*, vol. 36, no. 1, pp. 101–6, 2015.

[69] D. Naidoo, S. F. van Vuuren, R. L. van Zyl, and H. de Wet, “Plants traditionally used individually and in combination to treat sexually transmitted infections in northern Maputaland, South Africa: Antimicrobial activity and cytotoxicity,” *J. Ethnopharmacol.*, vol. 149, no. 3, pp. 656–667, Oct. 2013.

[70] Du Toit Loots, and Francois H. van der Westhuizen, L. Botes, et al., “Aloe ferox leaf gel phytochemical content, antioxidant capacity, and possible health benefits.,” *J. Agric. Food Chem.*, vol. 55, no. 17, pp. 6891–6, Aug. 2007.

[71] C. M. Tata, C. R. Sewani-Rusike, O. O. Oyedeji, et al., “Antihypertensive effects of the hydro-ethanol extract of Senecio serratuloides DC in rats,” *BMC Complement. Altern. Med.*, vol. 19, no. 1, p. 52, Dec. 2019.

[72] P. Lutgen, “Artemisia afra and hypertension,” *Pharm. Pharmacol. Int. J.*, vol. 7, no. 6, pp. 297–300, Dec. 2019.

[73] M. Stompor, “A Review on Sources and Pharmacological Aspects of Sakuranetin,” *Nutrients*, vol. 12, no. 2, p. 513, Feb. 2020.

[74] M. Heinrich, “Sesotho Plant & Animal Names & Plants used by the Basotho, Rodney Moffett. SUN Press, Bloemfontain, South Africa (2010), R. 200 (incl. VAT), ISBN:978-1-920383-08-4 (pb), 306 pp.,” *J. Ethnopharmacol.*, vol. 139, p. 679, 2012.

[75] D. O. Ochwang’i, C. N. Kimwele, J. A. Oduma, et al., “Medicinal plants used in treatment and management of cancer in Kakamega County, Kenya,” *J. Ethnopharmacol.*, vol. 151, no. 3, pp. 1040–1055, Feb. 2014.

[76] A. Maroyi, “Diversity of use and local knowledge of wild and cultivated plants in the Eastern Cape province, South Africa,” *J. Ethnobiol. Ethnomed.*, vol. 13, no. 1, p. 43, Dec. 2017.

[77] A. Hutchings, A. . Scott, G. Lewis, and A. . Cunningham, *Zulu Medicinal Plants: An Inventory*. 1996.

[78] I. Bello, M. W. Shehu, M. Musa, M. Zaini Asmawi, and R. Mahmud, “Kigelia africana (Lam.) Benth. (Sausage tree): Phytochemistry and pharmacological review of a quintessential African traditional medicinal plant,” *J. Ethnopharmacol.*, vol. 189, pp. 253–276, Aug. 2016.

[79] T. O. Ajayi, J. O. Moody, and C. S. Anthony, “Ethnobotanical Survey of Plants used in the Management of Hypertension in Ibadan North Local Government Area of Oyo State, Nigeria,” *Niger. J. Pharm. Res.*, vol. 15, no. 1, pp. 61–73, Jul. 2019.

[80] O. O. Akanni, S. E. Owumi, and O. A. Adaramoye, “In vitro studies to assess the antioxidative, radical scavenging and arginase inhibitory potentials of extracts from Artocarpus altilis, Ficus exasperate and Kigelia africana,” *Asian Pac. J. Trop. Biomed.*, vol. 4, pp. S492–S499, May 2014.

[81] M. S. Ali-Shtayeh, R. M. Jamous, R. M. Jamous, and N. M. Y. Salameh, “Complementary and alternative medicine (CAM) use among hypertensive patients in Palestine,” *Complement. Ther. Clin. Pract.*, vol. 19, no. 4, pp. 256–263, Nov. 2013.

[82] N. el I. Harrat, S. Louala, F. Bensalah, et al., “Anti-hypertensive, anti-diabetic, hypocholesterolemic and antioxidant properties of prickly pear nopalitos in type 2 diabetic rats fed a high-fat diet,” *Nutr. Food Sci.*, vol. 49, no. 3, pp. 476–490, May 2019.

[83] A. Chauhan, P. K. Sharma, P. Srivastava, N. Kumar, and R. Dudhe, “Plants Having Potential Antidiabetic Activity: A Review,” *Sch. Res. Libr. Der Pharm. Lett.*, vol. 2, no. 3, pp. 369–387, 2010.

[84] X. Wang, C. Zhou, X. Yang, D. Miao, and Y. Zhang, “De Novo Transcriptome Analysis of Warburgia ugandensis to Identify Genes Involved in Terpenoids and Unsaturated Fatty Acids Biosynthesis,” *PLoS One*, vol. 10, no. 8, p. e0135724, Aug. 2015.

[85] F. Schultz, G. Anywar, B. Wack, C. L. Quave, and L.-A. Garbe, “Ethnobotanical study of selected medicinal plants traditionally used in the rural Greater Mpigi region of Uganda,” *J. Ethnopharmacol.*, vol. 256, p. 112742, Jun. 2020.

[86] V. E. Madikane, S. Bhakta, A. J. Russell, et al., “Inhibition of mycobacterial arylamine N-acetyltransferase contributes to anti-mycobacterial activity of Warburgia salutaris,” *Bioorg. Med. Chem.*, vol. 15, no. 10, pp. 3579–3586, May 2007.

[87] T. Rabe and J. van Staden, “Isolation of an antibacterial sesquiterpenoid from Warburgia salutaris,” *J. Ethnopharmacol.*, vol. 73, no. 1–2, pp. 171–174, Nov. 2000.

[88] A. K. Ibrahim, M. M. Radwan, S. A. Ahmed, et al., “Microbial metabolism of cannflavin A and B isolated from Cannabis sativa,” *Phytochemistry*, vol. 71, no. 8–9, pp. 1014–1019, Jun. 2010.

[89] O. Olorunnisola, G. Bradley, A. J. Afolayan, et al., “Ethnobotanical information on plants used for the management of cardiovascular diseases in Nkonkobe Municipality, South Africa,” *J. Med. Plants Res.*, vol. 5, no. 17, pp. 4256–4260, 2011.

[90] F. Kassie, F. Darroudi, M. Kundi, R. Schulte-Hermann, and S. Knasmuller, “Khat (Catha edulis) consumption causes genotoxic effects in humans,” *Int. J. Cancer*, vol. 92, no. 3, pp. 329–332, May 2001.

[91] S. S. Semenya and M. J. Potgieter, “Kirkia wilmsii: A Bapedi treatment for hypertension,” *South African J. Bot.*, vol. 100, pp. 228–232, Sep. 2015.

[92] A. Thakur, Y. S. Chun, N. October, H. O. Yang, and V. Maharaj, “Potential of South African medicinal plants targeting the reduction of Aβ42 protein as a treatment of Alzheimer’s disease,” *J. Ethnopharmacol.*, vol. 231, pp. 363–373, Mar. 2019.

[93] G. I. Stafford, M. E. Pedersen, J. van Staden, and A. K. Jäger, “Review on plants with CNS-effects used in traditional South African medicine against mental diseases,” *J. Ethnopharmacol.*, vol. 119, no. 3, pp. 513–537, Oct. 2008.

[94] B. Yemane, G. Medhanie, and K. S. Reddy, “Survey of Some Common Medicinal Plants Used In Eritrean Folk Medicine,” *Int. J. Med. Plants*, vol. 112, pp. 865–876, 2018.

[95] G. Thakur, M. Bag, B. Sanodiya, et al., “Momordica balsamina: A Medicinal and Neutraceutical Plant for Health Care Management,” *Curr. Pharm. Biotechnol.*, vol. 10, no. 7, pp. 667–682, Nov. 2009.

[96] J. A. O. Ojewole, S. O. Adewole, and G. Olayiwola, “Hypoglycaemic and hypotensive effects of Momordica charantia Linn (Cucurbitaceae) whole-plant aqueous extract in rats.,” *Cardiovasc. J. S. Afr.*, vol. 17, no. 5, pp. 227–32, 2006.

[97] A. Poduri, D. L. Rateri, S. K. Saha, S. Saha, and A. Daugherty, “Citrullus lanatus ‘sentinel’ (watermelon) extract reduces atherosclerosis in LDL receptor-deficient mice,” *J. Nutr. Biochem.*, vol. 24, no. 5, pp. 882–886, May 2013.

[98] S. I. Abdelwahab, L. E. A. Hassan, H. M. Sirat, et al., “Anti-inflammatory activities of cucurbitacin E isolated from Citrullus lanatus var. citroides: Role of reactive nitrogen species and cyclooxygenase enzyme inhibition,” *Fitoterapia*, vol. 82, no. 8, pp. 1190–1197, Dec. 2011.

[99] S. Yadav, A. K. Tomar, O. Jithesh, et al., “Purification and Partial Characterization of Low Molecular Weight Vicilin-Like Glycoprotein from the Seeds of Citrullus lanatus,” *Protein J.*, vol. 30, no. 8, pp. 575–580, Dec. 2011.

[100] M. S. Association of Food Scientists & Technologists (India) and P. Ramakrishna, *Journal of food science and technology.*, vol. 21, no. 5. Association of Food Scientists & Technologists, India, 1984.

[101] R. O. Arise, A. A. Yekeen, O. E. Ekun, and O. J. Olatomiwa, “Protein Hydrolysates from Citrullus lanatus Seed: Antiradical and Hydrogen Peroxide-scavenging properties and kinetics of Angiotensin-I converting enzyme inhibition,” *Ceylon J. Sci.*, vol. 45, no. 2, p. 39, 2016.

[102] S. S. Semenya, M. J. Potgieter, and M. P. Tshisikhawe, “Use, conservation and present availability status of ethnomedicinal plants of Matebele-Village in the Limpopo Province, South Africa,” *African J. Biotechnol.*, vol. 12, no. 18, pp. 2392–2405, 2013.

[103] A. Gbolade, “Ethnobotanical study of plants used in treating hypertension in Edo State of Nigeria,” *J. Ethnopharmacol.*, vol. 144, no. 1, pp. 1–10, Oct. 2012.

[104] F. Bano, N. Akthar, and H. Naz, “Effect of the aqueous extract of momordica charantia On body weight of rats,” *J. Basic Appl. Sci.*, vol. 7, no. 1, pp. 1–5, 2011.

[105] K. K. Sampath and D. Bhowmik, “Traditional Medicinal Uses And Therapeutic Benefits Of Momordica Charantia Linn.,” *Int. J. Pharm. Sci. Rev. Res.*, vol. 4, no. 3, pp. 23–28, 2010.

[106] M. Gupta, S. Sharma, A. K. Gautam, and R. Bhadauria, “Momordica charantia linn. (karela): nature’s silent healer,” *Int. J. Pharm. Sci. Rev. Res.*, vol. 11, no. 1, pp. 32–37, 2011.

[107] R. Bipat, J. R. Toelsie, R. F. Joemmanbaks, et al., “Effects of plants popularly used against hypertension on norepinephrine-stimulated guinea pig atria,” *Pharmacogn. Mag.*, vol. 4, no. 13, pp. 12–19, 2005.

[108] S. Dinakaran, K. Sharathnath, P. Yogeswaran, et al., “A medicinal potency of Momordica charantia,” *Int. J. Pharm. Sci. Rev. Res.*, vol. 1, no. 2, pp. 95–100, 2010.

[109] D. A. Mulholland, V. Sewram, R. Osborne, K. H. Pegel, and J. D. Connolly, “Cucurbitane triterpenoids from the leaves of Momordica foetida,” *Phytochemistry*, vol. 45, no. 2, pp. 391–395, May 1997.

[110] R. Acquaviva, C. Di Giacomo, L. Vanella, et al., “Antioxidant activity of extracts of Momordica Foetida Schumach. et Thonn.,” *Molecules*, vol. 18, no. 3, pp. 3241–3249, Mar. 2013.

[111] P. J. Waako, B. Gumede, P. Smith, and P. I. Folb, “The in vitro and in vivo antimalarial activity of Cardiospermum halicacabum L. and Momordica foetida Schumch. Et Thonn.,” *J. Ethnopharmacol.*, vol. 99, no. 1, pp. 137–143, May 2005.

[112] M. van de Venter, S. Roux, L. C. Bungu, et al., “Antidiabetic screening and scoring of 11 plants traditionally used in South Africa,” *J. Ethnopharmacol.*, vol. 119, no. 1, pp. 81–6, Sep. 2008.

[113] M. S. Deutschländer, N. Lall, M. Van de Venter, and S. Dewanjee, “The hypoglycemic activity of Euclea undulata Thunb. var. myrtina (Ebenaceae) root bark evaluated in a streptozotocin–nicotinamide induced type 2 diabetes rat model,” *South African J. Bot.*, vol. 80, pp. 9–12, May 2012.

[114] M. S. S. Deutschländer, M. van de Venter, S. Roux, J. Louw, and N. Lall, “Hypoglycaemic activity of four plant extracts traditionally used in South Africa for diabetes,” *J. Ethnopharmacol.*, vol. 124, no. 3, pp. 619–624, Jul. 2009.

[115] M. H. A. Suleiman, “An ethnobotanical survey of medicinal plants used by communities of Northern Kordofan region, Sudan,” *J. Ethnopharmacol.*, vol. 176, pp. 232–242, Dec. 2015.

[116] R. Ilavarasan, M. Mallika, and S. Venkataraman, “Anti-inflammatory and free radical scavenging activity of Ricinus communis root extract.,” *J. Ethnopharmacol.*, vol. 103, no. 3, pp. 478–80, Feb. 2006.

[117] R. Ilavarasan, M. Mallika, and S. Venkataraman, “Toxicological assessment of *Ricinus communis* Linn root extracts,” *Toxicol. Mech. Methods*, vol. 21, no. 3, pp. 246–250, Mar. 2011.

[118] E. E. Quist, R. D. Phillips, and F. K. Saalia, “Angiotensin converting enzyme inhibitory activity of proteolytic digests of peanut (Arachis hypogaea L.) flour,” *LWT - Food Sci. Technol.*, vol. 42, no. 3, pp. 694–699, Apr. 2009.

[119] V. K. Jimsheena and L. R. Gowda, “Arachin derived peptides as selective angiotensin I-converting enzyme (ACE) inhibitors: Structure–activity relationship,” *Peptides*, vol. 31, no. 6, pp. 1165–1176, Jun. 2010.

[120] K. Sebei, A. Gnouma, W. Herchi, F. Sakouhi, and S. Boukhchina, “Lipids, proteins, phenolic composition, antioxidant and antibacterial activities of seeds of peanuts (Arachis hypogaea l) cultivated in Tunisia,” *Biol. Res.*, vol. 46, no. 3, pp. 257–263, 2013.

[121] S. Mpofu, D. Tantoh Ndinteh, S. F. van Vuuren, D. K. Olivier, and R. W. M. Krause, “Interactive efficacies of Elephantorrhiza elephantina and Pentanisia prunelloides extracts and isolated compounds against gastrointestinal bacteria,” *South African J. Bot.*, vol. 94, pp. 224–230, Sep. 2014.

[122] S. J. Mpofu, T. A. M. M. Msagati, and R. W. M. M. Krause, “Cytotoxicity, phytochemical analysis and antioxidant activity of crude extracts from rhizomes of Elephantorrhiza elephantina and Pentanisia prunelloides.,” *Afr. J. Tradit. Complement. Altern. Med.*, vol. 11, no. 1, pp. 34–52, 2014.

[123] L. Seleteng Kose, A. Moteetee, and S. Van Vuuren, “Ethnobotanical survey of medicinal plants used in the Maseru district of Lesotho,” *J. Ethnopharmacol.*, vol. 170, pp. 184–200, Jul. 2015.

[124] S. Shaik, N. Singh, and A. Nicholas, “Comparison of the selected secondary metabolite content present in the cancer-bush lessertia (Sutherlandia) frutescens l. extracts,” *African J. Tradit. Complement. Altern. Med.*, vol. 8, no. 4, pp. 429–434, 2011.

[125] R. Gomathi, M. Vijipriya, and K. Usha, “Cardioprotective effect of ethanolic extract of Medicago sativa stem on isoproterenol induced myocardial infarction in Wistar Albino rats,” *Int. J. Pharm. Pharm. Sci.*, vol. 6, pp. 839–842, 2014.

[126] R. Martínez, G. Kapravelou, J. M. Porres, et al., “Medicago sativa L., a functional food to relieve hypertension and metabolic disorders in a spontaneously hypertensive rat model,” *J. Funct. Foods*, vol. 26, pp. 470–484, Oct. 2016.

[127] R. Kapel, A. Chabeau, J. Lesage, et al., “Production, in continuous enzymatic membrane reactor, of an anti-hypertensive hydrolysate from an industrial alfalfa white protein concentrate exhibiting ACE inhibitory and opioid activities,” *Food Chem.*, vol. 98, no. 1, pp. 120–126, Jan. 2006.

[128] D. A., “Antibacterial evaluation and phytochemical analysis of Medicago sativa L. against some Microbial pathogens,” *Indian J. Sci. Technol.*, vol. 4, pp. 550–552, 2011.

[129] G. S. Taïwe, “Neurotoxicity and Neuroprotective Effects of African Medicinal Plants,” *Toxicol. Surv. African Med. Plants*, pp. 423–444, Jan. 2014.

[130] L. Roux, M. Marianne, and others, “Trifolium africanum,” *SANBI*, 2018.

[131] S. van Vuuren and L. Frank, “Review: Southern African medicinal plants used as blood purifiers,” *J. Ethnopharmacol.*, vol. 249, p. 112434, Mar. 2020.

[132] G. J. Amabeoku, “Antidiarrhoeal activity of Geranium incanum Burm. f. (Geraniaceae) leaf aqueous extract in mice,” *J. Ethnopharmacol.*, vol. 123, no. 1, pp. 190–193, May 2009.

[133] P. Baskaran, S. Singh, and J. Van Staden, “In vitro propagation, proscillaridin A production and antibacterial activity in Drimia robusta,” *Plant Cell, Tissue Organ Cult.*, vol. 114, no. 2, pp. 259–267, Aug. 2013.

[134] R. P. Luyt, A. K. Jäger, and J. van Staden, “The rational usage of Drimia robusta Bak. in traditional medicine,” *South African J. Bot.*, vol. 65, no. 4, pp. 291–294, Aug. 1999.

[135] P. Baskaran, A. Kumari, and J. Van Staden, “Analysis of the effect of plant growth regulators and organic elicitors on antibacterial activity of Eucomis autumnalis and Drimia robusta ex vitro-grown biomass,” *Plant Growth Regul.*, vol. 85, no. 1, pp. 143–151, May 2018.

[136] B. Ncube, A. R. Ndhlala, A. Okem, and J. van Staden, “Hypoxis (Hypoxidaceae) in African traditional medicine,” vol. 150, no. 3, pp. 818–827, Dec. 2013.

[137] N. R. Sathekge, “Comparison of secondary metabolite content and antimicrobial activity of four Hypoxis species used in traditional medicine,” University of Pretoria, 2011.

[138] M. B. C. Simelane, “Antimalarial activity of hypoxis colchicifolia, bak, mimusops caffra, E. Mey. ex A.DC and mimusops obtusifolia, lam from South Africa,” University of Zululand, 2014.

[139] M. R. Appleton, “Micropropagation of Hypoxis colchicifolia Baker, a valuable medicinal plant.,” 2004.

[140] J. A. O. Ojewole, D. R. Kamadyaapa, and C. T. Musabayane, “Some in vitro and in vivo cardiovascular effects of Hypoxis hemerocallidea Fisch & CA Mey (Hypoxidaceae) corm (African potato) aqueous extract in experimental animal models.,” *Cardiovasc. J. South Africa Off. J. South. Africa Card. Soc. [and] South African Soc. Card. Pract.*, vol. 17, no. 4, pp. 166–171, 2006.

[141] K. Magwede, L. I. Ramovha, Ḓ.E.N. Mabogo, A. E. van Wyk, and B.-E. van Wyk, “Traditional uses of the remarkable root bark hairs of Lannea schweinfurthii var. stuhlmannii (Anacardiaceae) by the Vhavenḓa, South Africa,” *South African J. Bot.*, vol. 122, pp. 529–534, May 2019.

[142] S. Odeyemi and G. Bradley, “Medicinal Plants Used for the Traditional Management of Diabetes in the Eastern Cape, South Africa: Pharmacology and Toxicology,” *Molecules*, vol. 23, 2018.

[143] L. E. Moagi, P. J. Schutte, and C. M. L. Steinmann, “Cardiovascular effects of dietes iridioides in Spontaneous Hypertensive Rats (SHR),” University of Limpopo (Medunsa Campus), 2013.

[144] J. Pujol, *NaturAfrica : the herbalist handbook : African flora, medicinal plants*. Durban, Republic of South Africa: J. Pujol Natural Healers Foundation ;, 1990.

[145] R. Sarma, S. Kumari, R. Elancheran, M. Deori, and R. Devi, “Polyphenol Rich Extract of Garcinia pedunculata Fruit Attenuates the Hyperlipidemia Induced by High Fat Diet,” *Front. Pharmacol.*, vol. 7, p. 294, Aug. 2016.

[146] M. Niazi, F. Yari, and A. Shakarami, “A Review of Medicinal Herbs in the Lamiaceae Family Used to Treat Arterial Hypertension,” *Entomol. Appl. Sci. Lett.*, vol. 6, no. 1, pp. 22–27, 2019.

[147] B. N. Nsuala, G. Enslin, and A. Viljoen, “‘Wild cannabis’: A review of the traditional use and phytochemistry of Leonotis leonurus,” *J. Ethnopharmacol.*, vol. 174, pp. 520–539, Nov. 2015.

[148] V. Maphosa, P. Masika, and A. Adedapo, “Safety evaluation of the aqueous extract of Leonotis leonurus shoots in rats.,” *Hum. Exp. Toxicol.*, vol. 27, no. 11, pp. 837–43, Nov. 2008.

[149] K. Obikeze, P. Mugabo, I. Green, et al., “Effects of a Methanol Fraction of the Leaves of Leonotis leonurus on the Blood Pressure and Heart Rate of Normotensive Male Wistar Rats,” vol. 7, no. 9, pp. 525–528, 2013.

[150] A. Venditti, C. Frezza, D. Celona, et al., “Phytochemical comparison with quantitative analysis between two flower phenotypes of Mentha aquatica L .: pink-violet and white,” vol. 4, no. August, pp. 288–300, 2017.

[151] M. S. Akhtar, Q. Jabeen, S. Bashir, et al., “Antihypertensive and toxicity studies of aqueous methanolic extract of mentha longifolia L .,” vol. 23, no. 6, pp. 1622–1627, 2013.

[152] J. J. J. De Beer and B.-E. Van Wyk, “An ethnobotanical survey of the Agter–Hantam, Northern Cape Province, South Africa,” *South African J. Bot.*, vol. 77, no. 3, pp. 741–754, Aug. 2011.

[153] A. Umar, G. Imam, W. Yimin, et al., “Antihypertensive effects of Ocimum basilicum L. (OBL) on blood pressure in renovascular hypertensive rats,” *Hypertens. Res.*, vol. 33, no. 7, pp. 727–730, Jul. 2010.

[154] S. Amrani, H. Harnafi, D. Gadi, et al., “Vasorelaxant and anti-platelet aggregation effects of aqueous Ocimum basilicum extract,” *J. Ethnopharmacol.*, vol. 125, no. 1, pp. 157–162, Aug. 2009.

[155] E. A. Irondi, S. O. Agboola, G. Oboh, and A. A. Boligon, “Inhibitory effect of leaves extracts of Ocimum basilicum and Ocimum gratissimum on two key enzymes involved in obesity and hypertension in vitro.,” *J. Intercult. Ethnopharmacol.*, vol. 5, no. 4, pp. 396–402, 2016.

[156] T. Tcheghebe, L. D. Nyamen, N. Tatong, and A. J. Seukep, “Ethnobotanical uses, phytochemical and pharmacological profiles, and toxicity of persea Americana mill.: An overview,” *Pharmacologyonline*, vol. 3, pp. 213–221, 2016.

[157] M. Yasir, S. Das, and M. D. Kharya, “The phytochemical and pharmacological profile of Persea americana Mill.,” *Pharmacogn. Rev.*, vol. 4, no. 7, pp. 77–84, Jan. 2010.

[158] B.-E. van Wyk, B. van Oudtshoorn, and N. Gericke, *Medicinal plants of South Africa*, 2nd Editio. Pretoria, South Africa: Briza Publications, 2013.

[159] Q. Wu, Q. Huang, Z. Chen, et al., “Effects of Cinnamomum Camphora Forest Environment on Elderly Patients with Hypertension: Implications for Adjunctive Therapy,” in *Proceedings of the 2nd Symposium on Health and Education 2019 (SOHE 2019)*, 2019, pp. 203–209.

[160] G. Chen, H. Zhang, and J. Ye, “Determination of rutin and quercetin in plants by capillary electrophoresis with electrochemical detection,” *Anal. Chim. Acta*, vol. 423, no. 1, pp. 69–76, Sep. 2000.

[161] J. Fu, C. Zeng, Z. Zeng, B. Wang, and D. Gong, “*Cinnamomum camphora* Seed Kernel Oil Ameliorates Oxidative Stress and Inflammation in Diet-Induced Obese Rats,” *J. Food Sci.*, vol. 81, no. 5, pp. H1295–H1300, May 2016.

[162] D. Prashanth, M. K. Asha, and A. Amit, “Antibacterial activity of Punica granatum,” *Fitoterapia*, vol. 72, no. 2, pp. 171–173, Feb. 2001.

[163] E. P. Lansky and R. A. Newman, “Punica granatum (pomegranate) and its potential for prevention and treatment of inflammation and cancer,” *J. Ethnopharmacol.*, vol. 109, no. 2, pp. 177–206, Jan. 2007.

[164] M. Mohan, H. Waghulde, and S. Kasture, “Effect of pomegranate juice on Angiotensin II-induced hypertension in diabetic wistar rats,” *Phyther. Res.*, vol. 24, no. S2, pp. S196–S203, Jun. 2010.

[165] A. Maroyi, “Dombeya rotundifolia (Hochst.) Planch.: review of its botany, medicinal uses, phytochemistry and biological activities,” *J. Complement. Med. Res.*, vol. 9, no. 3, p. 74, 2018.

[166] S. Ndwigah, G. Thoithi, J. Mwangi, and I. Kibwage, “Constituents of the stem bark of Dombeya rotundifolia Hochst,” *East Cent. African J. Pharm. Sci.*, vol. 8, no. 2, pp. 40–42, Nov. 2006.

[167] K. A. Reid, A. K. Jäger, and J. van Staden, “Pharmacological and phytochemical properties of Dombeya rotundifolia,” *South African J. Bot.*, vol. 67, no. 2, pp. 349–353, Jul. 2001.

[168] K. Konaté, K. Yomalan, O. Sytar, et al., “Free Radicals Scavenging Capacity, Antidiabetic and Antihypertensive Activities of Flavonoid-Rich Fractions from Leaves of *Trichilia emetica* and *Opilia amentacea* in an Animal Model of Type 2 Diabetes Mellitus,” *Evidence-Based Complement. Altern. Med.*, vol. 2014, pp. 1–13, 2014.

[169] D. Diallo, B. S. Paulsen, T. H. . Liljebäck, and T. E. Michaelsen, “The malian medicinal plant Trichilia emetica; studies on polysaccharides with complement fixing ability,” *J. Ethnopharmacol.*, vol. 84, no. 2–3, pp. 279–287, Feb. 2003.

[170] A. P. Djoupo, H. F. Yapi, G. Gnahoue, et al., “Phytochemical and Acute Toxicity Study of Trichilia Emetica (Meliaceaes) bark of trunk Extract in Albinos Rats,” *Am. J. Bio-pharmacology Biochem. Life Sci.*, vol. 4, no. 1, pp. 1–8, 2015.

[171] D. R. Kamadyaapa, M. M. Gondwe, K. Moodley, J. A. O. Ojewole, and C. T. Musabayane, “Cardiovascular effects of Ekebergia capensis Sparrm (Meliaceae) ethanolic leaf extract in experimental animal paradigms.,” *Cardiovasc. J. Afr.*, vol. 20, no. 3, pp. 162–7, 169, 2009.

[172] P. Singh, A. Mishra, P. Singh, S. Goswami, and A. Singh, “Hypertension and herbal plant for its treatment: a review,” *Indian J. Res. Pharm. Biotechnol.*, vol. 3, no. 5, pp. 358–366, 2015.

[173] C. T. Musabayane, “The effects of medicinal plants on renal function and blood pressure in diabetes mellitus.,” *Cardiovasc. J. Afr.*, vol. 23, no. 8, pp. 462–8, Sep. 2012.

[174] D. F. Fitzpatrick, S. L. Hirschfield, T. Ricci, P. Jantzen, and R. G. Coffey, “Endothelium-Dependent Vasorelaxation Caused by Various Plant Extracts,” *J. Cardiovasc. Pharmacol.*, vol. 26, no. 1, 1995.

[175] O. A. Fawole, S. O. Amoo, A. R. Ndhlala, et al., “Anti-inflammatory, anticholinesterase, antioxidant and phytochemical properties of medicinal plants used for pain-related ailments in South Africa,” *J. Ethnopharmacol.*, vol. 127, no. 2, pp. 235–241, Feb. 2010.

[176] R. Jahan, A. Khatun, N. Nahar, et al., “Use of Menispermaceae family plants in folk medicine of Bangladesh,” *Adv. Nat. Appl. Sci.*, vol. 4, no. 1, pp. 1–10, Jan. 2010.

[177] H. De Wet, F. Van Heerden, B. Van Wyk, R. V. Z.- Fitoterapia, and undefined 2007, “Antiplasmodial activity and cytotoxicity of Albertisia delagoensis,” *academia.edu*.

[178] J. O. Babajide, “Chemical and biological investigation into some selected African indigenous medicinal plants,” 2009.

[179] S. Ayers, D. L. Zink, K. Mohn, et al., “Anthelmintic activity of aporphine alkaloids from Cissampelos capensis.,” *Planta Med.*, vol. 73, no. 3, pp. 296–7, Mar. 2007.

[180] A. C. Cavalcanti, I. C. A. R. Melo, A. F. D. Medeiros, et al., “Studies with Cissampelos sympodialis: the search towards the scientific validation of a traditional Brazilian medicine used for the treatment of asthma,” *Rev. Bras. Farmacogn.*, vol. 23, no. 3, pp. 527–541, May 2013.

[181] N. S. Mathew and P. S. Negi, “Traditional uses, phytochemistry and pharmacology of wild banana (Musa acuminata Colla): A review,” *J. Ethnopharmacol.*, vol. 196, pp. 124–140, Jan. 2017.

[182] J. K. Muthee, D. W. Gakuya, J. M. Mbaria, et al., “Ethnobotanical study of anthelmintic and other medicinal plants traditionally used in Loitoktok district of Kenya,” *J. Ethnopharmacol.*, vol. 135, no. 1, pp. 15–21, Apr. 2011.

[183] Y. Liang, T. Li, G. Lu, et al., “Observation of Musa acuminate’s effect on hypertension patients with oxidatie stress disorders,” *China J. Tradit. Chinese Med. Pharm.*, vol. 7, pp. 1967–1969, 2012.

[184] Y. Liang, T. Li, G. Lu, J. Lan, and J. Li, “Experiments and research on the curative effect and mechanism of treatment to spontaneously hypertensive rats by Musa acuminate,” *Lishizhen Med. Mater. Medica Res.*, vol. 10, no. 5, pp. 2324–2326, 2013.

[185] T. E. Kwape, R. R. T. Majinda, and P. Chaturvedi, “Antioxidant and antidiabetic potential of Myrothamnus flabellifolius found in Botswana,” *Cogent Biol.*, vol. 2, no. 1, p. 1275403, Dec. 2016.

[186] Motlhanka D. M.T and G. Mathapa, “Antioxidant activities of crude extracts from medicinal plants used by diabetic patients in Eastern Botswana,” *J. Med. Plants Res.*, vol. 6, no. 42, pp. 5460–5463, Nov. 2012.

[187] J. O. Erhabor, R. Komakech, Y. Kang, M. Tang, and M. G. Matsabisa, “Ethnopharmacological importance and medical applications of Myrothamnus flabellifolius Welw. (Myrothamnaceae)-A review,” *J. Ethnopharmacol.*, vol. 252, p. 112576, Apr. 2020.

[188] L. S. Mhlongo and B.-E. Van Wyk, “Zulu medicinal ethnobotany: new records from the Amandawe area of KwaZulu-Natal, South Africa,” *South African J. Bot.*, vol. 122, pp. 266–290, May 2019.

[189] A. O. Ademiluyi, G. Oboh, O. B. Ogunsuyi, and F. M. Oloruntoba, “A comparative study on antihypertensive and antioxidant properties of phenolic extracts from fruit and leaf of some guava (Psidium guajava L.) varieties,” *Comp. Clin. Path.*, vol. 25, no. 2, pp. 363–374, Mar. 2016.

[190] C. K. Roy, J. V Kamath, and M. Asad, “Hepatoprotective activity of Psidium guajava Linn. leaf extract.,” *Indian J. Exp. Biol.*, vol. 44, no. 4, pp. 305–11, Apr. 2006.

[191] M. Ayub, M. Norazmir, S. Mamot, K. Jeeven, and H. Hadijah, “Anti-hypertensive effect of pink guava (Psidium guajava) puree on spontaneous hypertensive rats.,” *Int. Food Res. J.*, vol. 17, no. 1, pp. 89–96, 2010.

[192] V. Arya, N. Thakur, and C. P. Kashyap, “Preliminary Phytochemical Analysis of the Extracts of Psidium Leaves,” *J. Pharmacogn. Phytochem.*, vol. 1, no. 1, pp. 1–5, 2012.

[193] E. Díaz-de-Cerio, V. Verardo, A. M. Gómez-Caravaca, A. Fernández-Gutiérrez, and A. Segura-Carretero, “Health Effects of Psidium guajava L. Leaves: An Overview of the Last Decade.,” *Int. J. Mol. Sci.*, vol. 18, no. 4, Apr. 2017.

[194] J. A. Ojewole, “Hypoglycemic and hypotensive effects of Psidium guajava Linn. (Myrtaceae) leaf aqueous extract.,” *Methods Find. Exp. Clin. Pharmacol.*, vol. 27, no. 10, pp. 689–95, Dec. 2005.

[195] R. M. P. Gutiérrez, S. Mitchell, and R. V. Solis, “Psidium guajava: a review of its traditional uses, phytochemistry and pharmacology.,” *J. Ethnopharmacol.*, vol. 117, no. 1, pp. 1–27, Apr. 2008.

[196] R. B. Singh, S. S. Rastogi, N. K. Singh, et al., “Can guava fruit intake decrease blood pressure and blood lipids?,” *J. Hum. Hypertens.*, vol. 7, no. 1, pp. 33–8, Feb. 1993.

[197] E. A. Irondi, S. O. Agboola, G. Oboh, et al., “Guava leaves polyphenolics-rich extract inhibits vital enzymes implicated in gout and hypertension in vitro.,” *J. Intercult. Ethnopharmacol.*, vol. 5, no. 2, pp. 122–130, 2016.

[198] J. Mensah, R. Okoli, A. Turay, and E. Ogie-Odia, “Phytochemical Analysis of Medicinal Plants Used for the Management of Hypertension by Esan people of Edo State, Nigeria,” *Ethnobot. Leafl.*, vol. 2009, no. 10, Oct. 2009.

[199] N. Md Nor and A. Yatim, “Effects of Pink Guava (Psidium guajava) Puree Supplementation on Antioxidant Enzyme Activities and Organ Function of Spontaneous Hypertensive Rat,” *Sains Malaysiana*, vol. 40, pp. 369–372, 2011.

[200] M. A. Hashmi, A. Khan, M. Hanif, U. Farooq, and S. Perveen, “Traditional Uses, Phytochemistry, and Pharmacology of *Olea europaea* (Olive),” *Evidence-Based Complement. Altern. Med.*, vol. 2015, pp. 1–29, 2015.

[201] L. Somova, F. Shode, P. Ramnanan, and A. Nadar, “Antihypertensive, Antiatherosclerotic and Antioxidant Activity of Triterpenoids Isolated From Olea Europaea, Subspecies Africana Leaves,” *J. Ethnopharmacol.*, vol. 84, no. 2–3, 2003.

[202] N. Bennani-Kabchi, H. Fdhil, Y. Cherrah, et al., “[Therapeutic effect of Olea europea var. oleaster leaves on carbohydrate and lipid metabolism in obese and prediabetic sand rats (Psammomys obesus)].,” *Ann. Pharm. Fr.*, vol. 58, no. 4, pp. 271–277, Jul. 2000.

[203] O. Benavente-García, J. Castillo, J. Lorente, and M. Alcaraz, “Radioprotective Effects *In Vivo* of Phenolics Extracted from *Olea europaea* L. Leaves Against X-Ray-Induced Chromosomal Damage: Comparative Study Versus Several Flavonoids and Sulfur-Containing Compounds,” *J. Med. Food*, vol. 5, no. 3, pp. 125–135, Sep. 2002.

[204] A. D. Crawford, S. Liekens, A. R. Kamuhabwa, et al., “Zebrafish Bioassay-Guided Natural Product Discovery: Isolation of Angiogenesis Inhibitors from East African Medicinal Plants,” *PLoS One*, vol. 6, no. 2, p. e14694, Feb. 2011.

[205] S. Dehdari and H. Hajimehdipoor, “Medicinal Properties of Adiantum capillus-veneris Linn. in Traditional Medicine and Modern Phytotherapy: A Review Article.,” *Iran. J. Public Health*, vol. 47, no. 2, pp. 188–197, Feb. 2018.

[206] F. Haq, H. Ahmad, and M. Alam, *Traditional uses of medicinal plants of Nandiar Khuwarr catchment (District Battagram), Pakistan*, vol. 5, no. 1. Academic Journals, 2011.

[207] R. S. Aziz and Kawadizaye, “Diuretic effect of adiantumcapillus and its chemical constituents in hypertensive rats,” *Int. J. Pharm. Res.*, vol. 11, no. 3, pp. 111–119, 2019.

[208] J.-T. Chiang, K. Badrealam, M. Shibu, et al., “Anti-Apoptosis and Anti-Fibrosis Effects of Eriobotrya Japonica in Spontaneously Hypertensive Rat Hearts,” *Int. J. Mol. Sci.*, vol. 19, no. 6, p. 1638, May 2018.

[209] Y. Huang, J. Li, Q. Cao, et al., “Anti-oxidative effect of triterpene acids of Eriobotrya japonica (Thunb.) Lindl. leaf in chronic bronchitis rats,” *Life Sci.*, vol. 78, no. 23, pp. 2749–2757, May 2006.

[210] A. O. Aremu, O. A. Fawole, J. C. Chukwujekwu, et al., “In vitro antimicrobial, anthelmintic and cyclooxygenase-inhibitory activities and phytochemical analysis of Leucosidea sericea,” *J. Ethnopharmacol.*, vol. 131, no. 1, pp. 22–27, Aug. 2010.

[211] N. S. Mthethwa, B. A. Oyedeji, L. C. Obi, and O. A. Aiyegoro, “Anti-staphylococcal, anti-HIV and cytotoxicity studies of four South African medicinal plants and isolation of bioactive compounds from Cassine transvaalensis (Burtt. Davy) codd,” *BMC Complement. Altern. Med.*, vol. 14, no. 1, p. 512, Dec. 2014.

[212] F. N. Makhubu, “Isolation of bioactive compounds and in vitro studies on Pentanisia prunelloides (Klotzsch ex Eckl. &amp; Zeyh.) Walp. used in the eastern Free State for the management of diabetes mellitus,” 2017.

[213] E. Muleya, A. S. Ahmed, A. M. Sipamla, F. M. Mtunzi, and W. Mutatu, “Pharmacological properties of Pomaria sandersonii, Pentanisia prunelloides and Alepidea amatymbica extracts using in vitro assays,” *J. Pharmacogn. Phyther.*, vol. 7, no. 1, pp. 1–6, Jan. 2015.

[214] M. H. Alu’datt, T. Rababah, M. N. Alhamad, et al., “Profiles of free and bound phenolics extracted from Citrus fruits and their roles in biological systems: content, and antioxidant, anti-diabetic and anti-hypertensive properties,” *Food Funct.*, vol. 8, no. 9, pp. 3187–3197, Sep. 2017.

[215] B. Gürdal and Ş. Kültür, “An ethnobotanical study of medicinal plants in Marmaris (Muğla, Turkey),” *J. Ethnopharmacol.*, vol. 146, no. 1, pp. 113–126, Mar. 2013.

[216] G. Oboh, T. A. Olasehinde, and A. O. Ademosun, “Inhibition of enzymes linked to type-2 diabetes and hypertension by essential oils from peels of orange and lemon,” *Int. J. Food Prop.*, vol. 20, no. sup1, pp. S586–S594, Dec. 2017.

[217] J. Sidana, V. Saini, S. Dahiya, P. Nain, and S. Bala, “A Review on Citrus – ‘The Boon of Nature,’” *Int. J. Pharm. Sci. Rev. Res.*, vol. 18, no. 2, pp. 20–27, 2013.

[218] G. Oboh, F. O. Bello, and A. O. Ademosun, “Hypocholesterolemic properties of grapefruit (Citrus paradisii) and shaddock (Citrus maxima) juices and inhibition of angiotensin-1-converting enzyme activity,” *J. Food Drug Anal.*, vol. 22, no. 4, pp. 477–484, Dec. 2014.

[219] P. Vijaylakshmi and R. Radha, “An overview: Citrus maxima,” *J. Phytopharm.*, vol. 4, no. 5, pp. 263–267, 2015.

[220] H. Iwasaki, H. Oku, R. Takara, et al., “The tumor specific cytotoxicity of dihydronitidine from Toddalia asiatica Lam,” *Cancer Chemother. Pharmacol.*, vol. 58, no. 4, pp. 451–459, Oct. 2006.

[221] A. Kimang’a, J. Gikunju, D. Kariuki, and M. Ogutu, “Safety and analgesic properties of ethanolic extracts of Toddalia asiatica (L) Lam. (rutaceae) used for central and peripheral pain management among the east african ethnic communities,” *Ethiop. J. Health Sci.*, vol. 26, no. 1, p. 55, Jan. 2016.

[222] H. Kariuki, T. Kanui, A. Yenesew, N. Patel, and P. Mbugua, “Antinocieptive and anti-inflammatory effects of Toddalia asiatica (L) Lam. (Rutaceae) root extract in Swiss albino mice,” *Pan Afr. Med. J.*, vol. 14, 2013.

[223] N. Tabassum and F. Ahmad, “Role of natural herbs in the treatment of hypertension.,” *Pharmacogn. Rev.*, vol. 5, no. 9, pp. 30–40, Jan. 2011.

[224] A. Moolla and A. M. Viljoen, “‘Buchu’ – Agathosma betulina and Agathosma crenulata (Rutaceae): A review,” *J. Ethnopharmacol.*, vol. 119, no. 3, pp. 413–419, Oct. 2008.

[225] J. A. O. Ojewole, “Hypoglycaemic effect of Clausena anisata (Willd) Hook methanolic root extract in rats,” *J. Ethnopharmacol.*, vol. 81, no. 2, pp. 231–237, Jul. 2002.

[226] M. T. L. Ntamo, J. S. Paul, H. Leon, B. ouml hmer Linde, and M. G. Melvin, “The effects of an aqueous leaf extract of Clausena anisata (Willd.) Hook.f.ex Benth. on blood pressure, urine output, angiotensin II levels and cardiac parameters in spontaneously hypertensive rats,” *J. Med. Plants Res.*, vol. 10, no. 28, pp. 425–434, Jul. 2016.

[227] A. A. Ismail, B. A. Ahmad, A. Mohamed, et al., “A review of traditional uses, phytochemical and pharmacological aspects of selected members of Clausena genus (Rutaceae),” *J. Med. Plants Res.*, vol. 6, no. 38, pp. 5107–5118, Oct. 2012.

[228] J. Pujol, “Naturafrica,” in *The Herbalist Handbook*, Durban, South Africa: Jean Pujol Natural Healers’ Foundation, 1990.

[229] D. Mulholland, N. Crouch, P. Coombes, J. Magadula, and M. Randrianarivelojosia, “Unusual triterpenoids from African medicinal plants,” in *11th NAPRECA Symposium Book of Proceedings, Antananarivo, Madagascar*, 2006, pp. 20–26.

[230] C. Sewani-Rusike and M. Mammen, “Medicinal Plants Used as Home Remedies: A Family Survey by First Year Medical Students,” *African J. Tradit. Complement. Altern. Med.*, vol. 11, no. 5, p. 67, Oct. 2014.

[231] K. W. Chiu and A. Y. L. Fung, “The hypotensive effects of green bean (Phaseolus aureus), common rue (Ruta graveolens) and kelp (Laminaria japonica) in rats,” *Phyther. Res.*, vol. 11, no. 3, pp. 203–206, May 1997.

[232] K. Lingaraju, H. Raja Naika, K. Manjunath, et al., “Biogenic synthesis of zinc oxide nanoparticles using Ruta graveolens (L.) and their antibacterial and antioxidant activities,” *Appl. Nanosci.*, vol. 6, no. 5, pp. 703–710, Jun. 2016.

[233] M. Ratheesh, G. L. Shyni, G. Sindhu, and A. Helen, “Inhibitory effect of Ruta graveolens L. on oxidative damage, inflammation and aortic pathology in hypercholesteromic rats,” *Exp. Toxicol. Pathol.*, vol. 63, no. 3, pp. 285–290, Mar. 2011.

[234] I. J. Fernández, P. N. Gómez, J. Parodi, F. R. Mejía, and R. S. Salazar, “Chilean crude extract of Ruta graveolens generates vasodilatation in rat aorta at cellular subtoxic concentrations,” *Adv. Biosci. Biotechnol.*, vol. 04, no. 01, pp. 29–36, Jan. 2013.

[235] M. R. Sailani and H. Moeini, “Effect of Ruta graveolens and Cannabis sativa alcoholic extract on spermatogenesis in the adult wistar male rats.,” *Indian J. Urol.*, vol. 23, no. 3, pp. 257–60, Jul. 2007.

[236] N. A. Jaradat, B. Damiri, and M. N. Abualhasan, “Antioxidant evaluation for Urtica urens, Rumex cyprius and Borago officinalis edible wild plants in Palestine,” *Pak. J. Pharm. Sci.*, vol. 29, no. 1, pp. 325–330, 2016.

[237] F. Universidad de Costa Rica., A. Adedapo, A. Aliero, and A. Afolayan, *Revista de biología tropical.*, vol. 58, no. 4. Universidad de Costa Rica, 2010.

[238] S. Arslan, G. Terzioglu, S. Elcil, H. Deligoz, and A. Sen, “Assessing of anti-inflammatory effect of Small nettle’ (Urtica urens) increasing polarity extracts,” *J. Neuroimmunol.*, vol. 275, no. 1–2, p. 135, Oct. 2014.

[239] Z. Doukkali, K. Taghzouti, E. H. Bouidida, et al., “Evaluation of anxiolytic activity of methanolic extract of Urtica urens in a mice model,” *Behav. Brain Funct.*, vol. 11, no. 1, p. 19, Dec. 2015.

[240] N. A. Masondo, G. I. Stafford, A. O. Aremu, and N. P. Makunga, “Acetylcholinesterase inhibitors from southern African plants: An overview of ethnobotanical, pharmacological potential and phytochemical research including and beyond Alzheimer’s disease treatment,” *South African J. Bot.*, vol. 120, pp. 39–64, Jan. 2019.

[241] J. F. Kamanula, S. R. Belmain, D. R. Hall, et al., “Chemical variation and insecticidal activity of Lippia javanica (Burm. f.) Spreng essential oil against Sitophilus zeamais Motschulsky,” *Ind. Crops Prod.*, vol. 110, pp. 75–82, Dec. 2017.

[242] D. K. Olivier, E. A. Shikanga, S. Combrinck, et al., “Phenylethanoid glycosides from Lippia javanica,” *South African J. Bot.*, vol. 76, no. 1, pp. 58–63, Jan. 2010.

[243] A. Maroyi, “*Lippia javanica* (Burm.f.) Spreng.: Traditional and Commercial Uses and Phytochemical and Pharmacological Significance in the African and Indian Subcontinent,” *Evidence-Based Complement. Altern. Med.*, vol. 2017, pp. 1–34, 2017.

[244] V. K. Matta, P. K. Pasala, S. Netala, S. Pandrinki, and P. Konduri, “Anti Hypertensive Activity of the Ethanolic Extract of Lantana camara leaves on high salt loaded wistar albino rats,” *Pharmacogn. J.*, vol. 7, no. 5, pp. 289–295, Jul. 2015.

[245] K. Taoubi, M. Fauvel, J. Gleye, C. Moulis, and I. Fourasté, “Phenylpropanoid Glycosides from *Lantana camara* and *Lippia multiflora*,” *Planta Med.*, vol. 63, no. 02, pp. 192–193, Apr. 1997.

[246] M. Saxena, J. Saxena, and S. Khare, “A brief review on: Therapeutical values of Lantana camara plant.: Discovery Service for UNISA,” *Int. J. Pharm. Life Sci.*, vol. 3, no. 3, pp. 1551–1554, 2012.

[247] M. Tchicaillat-Landou, J. Petit, C. Gaiani, et al., “Ethnobotanical study of medicinal plants used by traditional healers for the treatment of oxidative stress-related diseases in the Congo Basin,” *J. Herb. Med.*, vol. 13, pp. 76–90, Sep. 2018.

[248] E. L. Ghisalberti, “Lantana camara L. (Verbenaceae),” *Fitoterapia*, vol. 71, no. 5, pp. 467–486, Sep. 2000.

[249] G. Murtaza, M. Mukhtar, and A. Sarfraz, “A Review: Antifungal Potentials of Medicinal Plants,” *J. Bioresour. Manag.*, vol. 2, no. 2, Jun. 2015.

[250] J. Lin, A. . Opoku, M. Geheeb-Keller, et al., “Preliminary screening of some traditional zulu medicinal plants for anti-inflammatory and anti-microbial activities,” *J. Ethnopharmacol.*, vol. 68, no. 1–3, pp. 267–274, Dec. 1999.

[251] A. R. Opoku, N. F. Maseko, and S. E. Terblanche, “The in vitro antioxidative activity of some traditional Zulu medicinal plants,” *Phyther. Res.*, vol. 16, no. S1, pp. 51–56, Mar. 2002.

[252] S. W. Odeyemi and A. J. Afolayan, “Identification of Antidiabetic Compounds from Polyphenolic-rich Fractions of Bulbine abyssinica A . Rich Leaves,” *Pharmacognosy Res.*, vol. 10, no. 1, pp. 72–80, 2018.

[253] R. Osborne, A. Grove, P. Oh, et al., “The magical and medicinal usage of Stangeria eriopus in South Africa,” *J. Ethnopharmacol.*, vol. 43, no. 2, pp. 67–72, Jul. 1994.

[254] B. Meurer-Grimes and D. W. Stevenson, “The biflavones of the cycadales revisited: Biflavones in Stangeria eriopus, Chigua restrepoi and 32 other species of cycadales,” *Biochem. Syst. Ecol.*, vol. 22, no. 6, pp. 595–603, Sep. 1994.
